# Autonomic Network Signatures of Interictal Cardiorespiratory Dysfunction in Patients With Temporal Lobe Epilepsy

**DOI:** 10.1212/WNL.0000000000218470

**Published:** 2026-09-03

**Authors:** Haatef Pourmotabbed, Derek J. Doss, Lucas E. Sainburg, William P. Nobis, Dario J. Englot, Victoria Morgan, Catie Chang

**Affiliations:** 1 Department of Biomedical Engineering, Vanderbilt University, Nashville, TN; 2 Vanderbilt University Institute of Imaging Science, Vanderbilt University Medical Center, Nashville, TN; 3 Vanderbilt Institute for Surgery and Engineering, Vanderbilt University, Nashville, TN; 4 Department of Neurology, Vanderbilt University Medical Center, Nashville, TN; 5 Department of Neurological Surgery, Vanderbilt University Medical Center, Nashville, TN; 6 Department of Radiology and Radiological Sciences, Vanderbilt University Medical Center, Nashville, TN; 7 Department of Electrical and Computer Engineering, Vanderbilt University, Nashville, TN.

## Abstract

**Background and Objectives:**

Temporal lobe epilepsy (TLE) co-occurs with interictal impairments in autonomic regulation and respiratory chemoreception, which have been related to higher risk of sudden unexpected death in epilepsy (SUDEP). Studies indicate that seizures lead to reorganization of central autonomic circuits extending beyond the temporal lobe. However, the relationship between brain network disruptions and interictal cardiorespiratory dysfunction remains largely unknown. The goal of this work was to test whether central autonomic network alterations underlie interictal cardiorespiratory dysfunction and higher SUDEP risk.

**Methods:**

We included simultaneous fMRI and peripheral physiology from patients with drug-resistant unilateral TLE and healthy controls. We used mediation and correlation analyses to relate atypical functional connectivity strength and structural volume of central autonomic regions in TLE to alterations of high-frequency and low-frequency heart rate variability (HF-HRV/LF-HRV), breathing rate (BR), and respiratory rate variability (RRV). Multivariate models were then used to investigate associations with disease variables and risk factors of SUDEP. Finally, we analyzed a subcohort of patients with postsurgical data and examined whether autonomic connectivity changes following surgery impact cardiorespiratory function.

**Results:**

Patients with TLE (n = 72, 49% female, 40.0 ± 12.8 years) exhibited widespread reductions in autonomic connectivity and volume compared with controls (n = 105, 53% female, 36.7 ± 12.8 years) (*p*_*FDR*_ < 0.05). Reduced connectivity mediated higher BR, lower RRV, and lower LF-HRV in TLE relative to controls (|*z*| = 2.14 to 5.28, *p*_FDR_ < 0.05). Left seizure laterality was independently related to reduced LF-HRV and RRV (Cohen’s *f* = 0.42, 0.45; *p*_FDR_ = 0.0055, 0.0046), and canonical correlation analysis linked atrophy in autonomic regions to generalized seizure occurrence and earlier age at onset (*r* = 0.61, *p*_FWER_ = 0.018). In the postsurgical subcohort (n = 34), autonomic connectivity alterations after surgery were positively correlated with changes in LF-HRV and RRV (ρ = 0.52, 0.41; *p*_FDR_ = 0.0076, 0.029).

**Discussion:**

Our results suggest that reorganization of the central autonomic network may contribute to chronic autonomic and interoceptive deficits in TLE. Mapping these brain network disruptions may help guide novel strategies to restore cardiorespiratory function. Validation in SUDEP cases is needed to establish clinical utility as biomarkers of SUDEP.

## Introduction

Temporal lobe epilepsy (TLE) is the most common type of epilepsy,^1^ and patients with TLE experience abnormal cardiac, respiratory, and autonomic function during both the ictal and interictal periods.^2,3^ For instance, temporal lobe seizures co-occur with apnea, arrhythmia, hypoxemia, and elevated blood pressure.^2^ Interictally, patients with TLE exhibit reduced heart rate variability (HRV), baroreceptor reflex sensitivity, and hypercapnic respiratory response, which are indices of chronic dysfunction in autonomic control, blood pressure regulation, and CO_2_ chemoreception.^3–5^ Importantly, strong evidence suggests that seizure-induced cardiorespiratory depression and chronic autonomic abnormalities play a major role in sudden unexpected death in epilepsy (SUDEP), one of the leading causes of epilepsy-related mortality.^6–8^

A structurally interconnected set of brain regions, referred to as the central autonomic network, is implicated in interoception and cardiorespiratory regulation.^2,9^ Seizures can disrupt brainstem cardiorespiratory control centers, either through mesial temporal structural pathways^10–12^ or spreading depolarization during generalization.^13^ Furthermore, patients with TLE present with widespread structural and functional alterations in autonomic regions even outside the temporal lobe.^14–17^ These observations indicate that the progressive effects of seizures lead to long-term reorganization of neuroregulatory brain networks,^16,17^ potentially resulting in interictal autonomic and interoceptive deficits linked to elevated SUDEP risk.^7,8^ However, the relationship between chronic brain network disruptions and interictal cardiorespiratory dysfunction in epilepsy remains largely unknown.

In this work, we hypothesize that functional and structural alterations within the central autonomic network underlie interictal cardiorespiratory dysfunction and higher risk of SUDEP. To test this hypothesis, we leveraged simultaneous fMRI and peripheral physiologic data to relate atypical functional network strength and structural volume of central autonomic regions to abnormal cardiac autonomic and breathing function in TLE. We then investigated associations with disease variables and risk factors of SUDEP. Finally, we analyzed patients with postsurgical data as a model of network perturbation and examined whether brain network reorganization following surgery impacts cardiorespiratory function. Overall, this study reveals brain signatures of abnormal physiology in TLE, identifying biomarkers that can be investigated in future studies of SUDEP cases.

## Methods

### Participants

This study included patients with drug-resistant unilateral TLE who agreed to undergo research MRI acquisition and were evaluated for epilepsy surgery at Vanderbilt University Medical Center from 2012 to 2023. The inclusion criteria were age 18–70 years and a diagnosis of unilateral TLE based on clinical presurgical evaluations, consisting of structural MRI, long-term video EEG, seizure semiology, and interictal PET. We also recruited healthy controls aged 18–70 years with no history of neurologic disease. In addition, a subcohort of patients agreed to undergo research MRI acquisition at least 1 year following epilepsy surgery.

### Standard Protocol Approvals, Registrations, and Patient Consents

The protocol for this study was approved by the Vanderbilt University Institutional Review Board. Written informed consent was obtained from all participants.

### Data Collection

Each research MRI session consisted of 2 resting-state fMRI scans and a T1-weighted scan for brain volume estimation (eMethods).^16,17^ Respiratory belt and photoplethysmography (PPG) data were recorded simultaneously with fMRI. We visually inspected the data and excluded participants with excessive motion or poor-quality respiratory or PPG data.

### fMRI Preprocessing and Connectivity Analysis

The fMRI data were preprocessed with RETROICOR and regression of tissue-based nuisance signals to mitigate non-neural effects (eMethods).^18^ Mean signals were extracted from the presurgical fMRI data for 118 autonomic regions. Regions of interest (ROIs) were obtained from the Brainnetome atlas (100 ROIs), encompassing the ventromedial prefrontal cortex (orbitofrontal gyrus and frontal pole), insula, cingulate gyrus, amygdala, hippocampus, parahippocampal gyrus, fusiform gyrus, temporal pole, basal ganglia, and thalamus.^19^ We also included 10 brainstem arousal ROIs (eFigure 1),^20^ 4 basal forebrain ROIs,^21^ and 2 bed nucleus of the stria terminalis (BNST) and 2 hypothalamus ROIs.^22^ These ROIs were selected to include core regions of the central autonomic network,^2,9^ limbic/paralimbic structures involved in respiratory modulation,^12,23^ and neuroregulatory centers integrating arousal and autonomic function.^10,24^

Pearson correlation was used to estimate the functional connectivity (FC) between ROIs, and correlations were Fisher z-transformed to normalize the variance before averaging across the 2 fMRI scans per participant. We then estimated the node strength of each ROI within the autonomic subnetwork by computing the average of its FC to all other autonomic ROIs.^25^

### Measures of Interictal Cardiac and Respiratory Function

We investigated 2 measures of cardiac autonomic function: low-frequency and high-frequency HRV (LF-HRV/HF-HRV), along with 2 measures of respiratory function: breathing rate (BR) and respiratory rate variability (RRV). HF-HRV indexes rapid parasympathetic modulation of heart rate, while LF-HRV is influenced by both parasympathetic and sympathetic activity.^26^ BR measures overall ventilatory drive, and RRV reflects dynamic regulation of breathing.^27^ Reduced interictal LF-HRV and HF-HRV in epilepsy have been linked to increased risk of SUDEP,^7,28^ and abnormal BR and RRV may be associated with more severe respiratory depression during seizures.^8,29,30^ Cardiorespiratory measures were derived from the PPG and respiratory data of each participant (eMethods).

### Statistical Comparisons of Autonomic Network Connectivity and Cardiorespiratory Function

We first used 2-sample *t* tests to investigate how autonomic FC strength and cardiorespiratory measures are altered in TLE compared with controls. We then performed 3 complementary analyses to examine the relationship between autonomic network connectivity and cardiorespiratory function in TLE.

- Analysis 1 examined how strongly the connectivity of the autonomic ROIs is associated with cardiorespiratory function in TLE. This involved performing Spearman correlation tests between the FC and cardiorespiratory measures in TLE.
- Analysis 2 examined whether the relationship between autonomic connectivity and cardiorespiratory function is altered in TLE compared with controls. This involved fitting general linear models (GLMs) to test for moderation effects of group (TLE or control) on the relationship between FC and cardiorespiratory measures.
- Analysis 3 examined whether autonomic network dysfunction is an underlying factor contributing to abnormal cardiorespiratory function in TLE. This involved implementing bootstrapping (n = 1 × 10^5^) to test for mediation effects^31^ of FC on the relationship between group and cardiorespiratory measures.

Before the statistical comparisons, effects of age and sex on each cardiorespiratory and FC variable were modeled from the linear fit to the control data and regressed out of all the participants’ data. Since 2 different MRI scanners (of the same model) were used for data collection, the variability associated with scanner was also removed. The statistical analyses were performed in MATLAB R2020a, and significance was assessed at 2-tailed *p* < 0.05 with false discovery rate (FDR) correction.

### Brain Volumetric Analysis

In addition to the functional network analysis, we sought to evaluate whether disruptions of brain structure in TLE relate to cardiorespiratory dysfunction. CAT12 was used to perform region-based morphometry on the presurgical MRI data.^32^ The gray matter volume was estimated for all 254 ROIs of the Brainnetome atlas, basal forebrain, BNST, and hypothalamus. The sum of gray matter, white matter, and CSF volumes was calculated for the 10 brainstem ROIs because of the difficulty in using conventional 3T MRI to resolve tissue types in the brainstem.^33^ An estimate of total intracranial volume (TIV) was also computed. The brain volume estimates were compared between TLE and controls (2-sample *t* tests) and related to the cardiorespiratory variables (moderation models and Spearman correlations) after adjusting for age, sex, and scanner. Previous studies suggest that adjusting for TIV may underestimate the effects of epilepsy on brain volume.^34^ Therefore, we performed the statistical analyses on the regional volume estimates both with and without TIV as an additional covariate.

### Association With Clinical Parameters of Disease Severity

We implemented multivariate models to investigate how autonomic network disruptions and cardiorespiratory dysfunction in TLE are associated with disease variables and risk factors of SUDEP. Five parameters were obtained from clinical assessments: age at epilepsy onset, epilepsy duration, total seizure frequency, presence of focal-to-bilateral tonic-clonic seizures (FBTCS), and seizure laterality. Earlier age at onset, longer epilepsy duration, and tonic-clonic seizure (TCS) occurrence are well-established risk factors of SUDEP.^35^ Patients with missing parameters were excluded from the models.

First, we fitted GLMs to test for independent effects of the 5 clinical parameters on each covariate-adjusted cardiorespiratory measure. A rank-based inverse normal transformation was applied to ensure normality of residuals. Variable selection was performed using Least Absolute Shrinkage and Selection Operator with leave-one-out cross validation,^36^ and *p*-values were FDR corrected. We also used Spearman correlations to examine the relationship between each pair of cardiorespiratory measures in TLE.

Next, canonical correlation analysis (CCA)^37^ was used to evaluate the association between the clinical parameters, cardiorespiratory measures, and autonomic FC strength. The CCA estimated latent modes that maximized the correlation between 2 sets of variables: a matrix *X* formed by the covariate-adjusted FC strength of all the autonomic ROIs and a matrix *Y* composed of the clinical parameters and covariate-adjusted cardiorespiratory measures. For each mode, this yields a correlation value along with a loading weight for each variable in *X* and *Y*. To improve the stability of the CCA, the dimensionality of *X* was first reduced by selecting the top principal components that explained ≥60% of the variance.^37^ The statistical significance of the correlations was evaluated using permutation tests (n = 1 × 10^5^) with familywise error rate (FWER) correction.^38^ The reliability of each loading weight was assessed using bootstrap *z*-scores (n = 1 × 10^5^).^37^ The same CCA procedure was then implemented to investigate the association between the volume of the autonomic ROIs (matrix *X*) and the clinical parameters and cardiorespiratory measures (matrix *Y*).

Further, we conducted sensitivity analyses to investigate moderation effects of 2 other clinical parameters on the relationship between autonomic FC strength and cardiorespiratory measures (eMethods). These parameters included number of antiepileptic drugs (AEDs) and MRI evidence of mesial temporal sclerosis.

### Postsurgical Changes in Autonomic Network Connectivity and Cardiorespiratory Function

We investigated whether brain network reorganization following surgery is associated with changes in cardiorespiratory function. We computed the FC node strength of ROIs within the autonomic subnetwork for both presurgical and post-surgical fMRI data after excluding right and left temporal lobe regions. FC and cardiorespiratory variables were adjusted for age, sex, and scanner. Spearman correlation tests were used to identify ROIs in which presurgical FC strength was related to each cardiorespiratory measure at *p* < 0.05 (FDR-corrected across ROIs). For both presurgical and postsurgical scans, we then computed mean values of the FC strength by averaging over all the identified ROIs for each cardiorespiratory measure.

Partial Spearman correlation tests were used to examine the relationship of the presurgical-to-postsurgical difference in each mean FC value with the presurgical-to-postsurgical difference in the corresponding cardiorespiratory measure while controlling for surgery type and time between surgery and postsurgical scan. We also used partial Spearman correlation models to examine independent effects of 3 clinical parameters (Engel seizure outcome at time of postsurgical scan, surgery type, and time between surgery and postsurgical scan) on the presurgical-to-postsurgical change in each mean FC and cardiorespiratory measure.

### Data Availability

MRI, demographic, and clinical data will be made available on Pennsieve (discover.pennsieve.io/). Derived data supporting the main findings will be made available on Github.

## Results

Participants included 77 patients with TLE and 110 controls. Seventy-four patients underwent epilepsy surgery, and 39 patients participated in postoperative MRI acquisition. After data quality assessments, the final cohort consisted of 72 patients with preoperative data, 34 patients with post-operative data, and 105 controls. Demographic and clinical variables are presented in Table 1 and compared between patient subgroups in eTables 1 and 2. FBTCS occurrence was unknown for 2 patients.

### Cardiorespiratory Function, Autonomic Network Connectivity, and Structural Volume in TLE vs Controls

**Figure 1:**
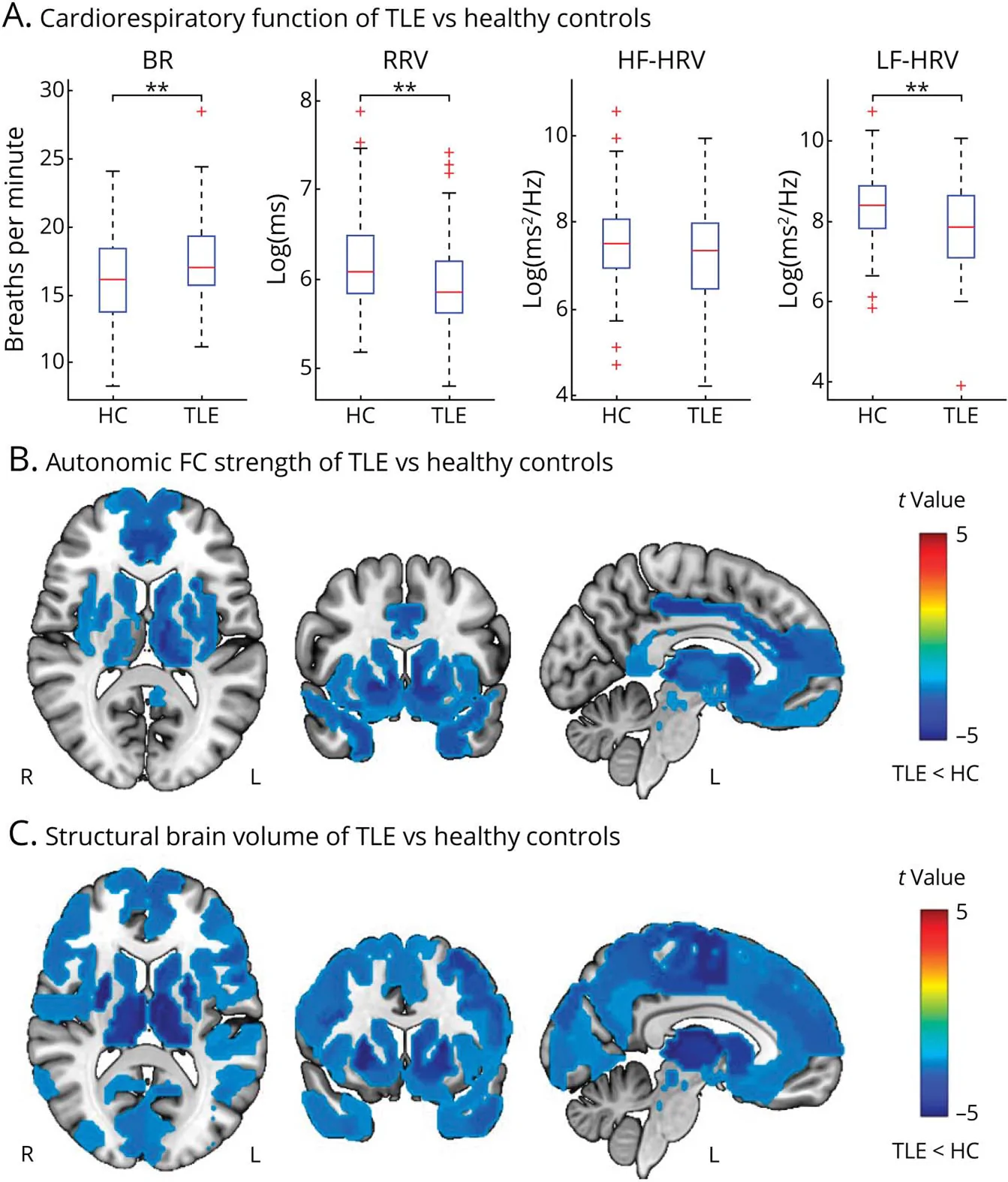
Abnormal Cardiorespiratory Function, Autonomic Network Connectivity, and Structural Volume in TLE (A) Comparison of the breathing rate (BR), respiratory rate variability (RRV), and high-frequency and low-frequency heart rate variability (HF-HRV/LF-HRV) between temporal lobe epilepsy (TLE) and healthy controls (HC). Two-sample *t* tests (2-tailed) were used for statistical comparisons. *p < 0.05, **p < 0.01, ***p < 0.001, false discovery rate (FDR)-corrected across 4 cardiorespiratory measures. (B) Regional differences in functional connectivity (FC) node strength of the central autonomic network between TLE and controls (*p* < 0.05, FDR-corrected across regions; 2-sample *t* tests, 2-tailed). (C) Regional differences in structural volume between TLE and controls (*p* < 0.05, FDR-corrected across regions; 2-sample *t* tests, 2-tailed). The FC, cardiorespiratory, and brain volume estimates were adjusted for age and sex. The brain volume estimates were not adjusted for total intracranial volume.

Patients with TLE exhibited abnormal cardiorespiratory function, as indicated by higher BR, lower RRV, and lower LF-HRV compared with controls (Cohen’s *d* = 0.54 for BR, −0.49 for RRV, and −0.47 for LF-HRV; *p*_FDR_ < 0.05; Figure 1A). Although nonsignificant, HF-HRV was also reduced in TLE (Cohen’s *d* = −0.20; *p*_FDR_ = 0.19). In addition, the FC node strength of the patients was reduced relative to controls in widespread areas of the central autonomic network (92 of 118 ROIs; *p*_FDR_ < 0.05; Figure 1B and eTable 3).

The brain volumetric analysis revealed that TIV was smaller in TLE compared with controls (Cohen *d* = −0.37; *p* = 0.017; eFigure 2A). Without adjusting for TIV, the patients exhibited volume reductions relative to controls in widespread brain areas (164 of 264 ROIs; *p*_FDR_ < 0.05; Figure 1C). Seventy-four of these ROIs were part of the autonomic subnetwork (eTable 4). After adjusting for TIV, the patients exhibited reduced volume compared with controls in 54 ROIs (*p*_FDR_ < 0.05; eFigure 2B), 32 of which belonged to the autonomic subnetwork (eTable 4).

### Associations of Autonomic Network Connectivity With Cardiorespiratory Function in TLE

**Figure 2:**
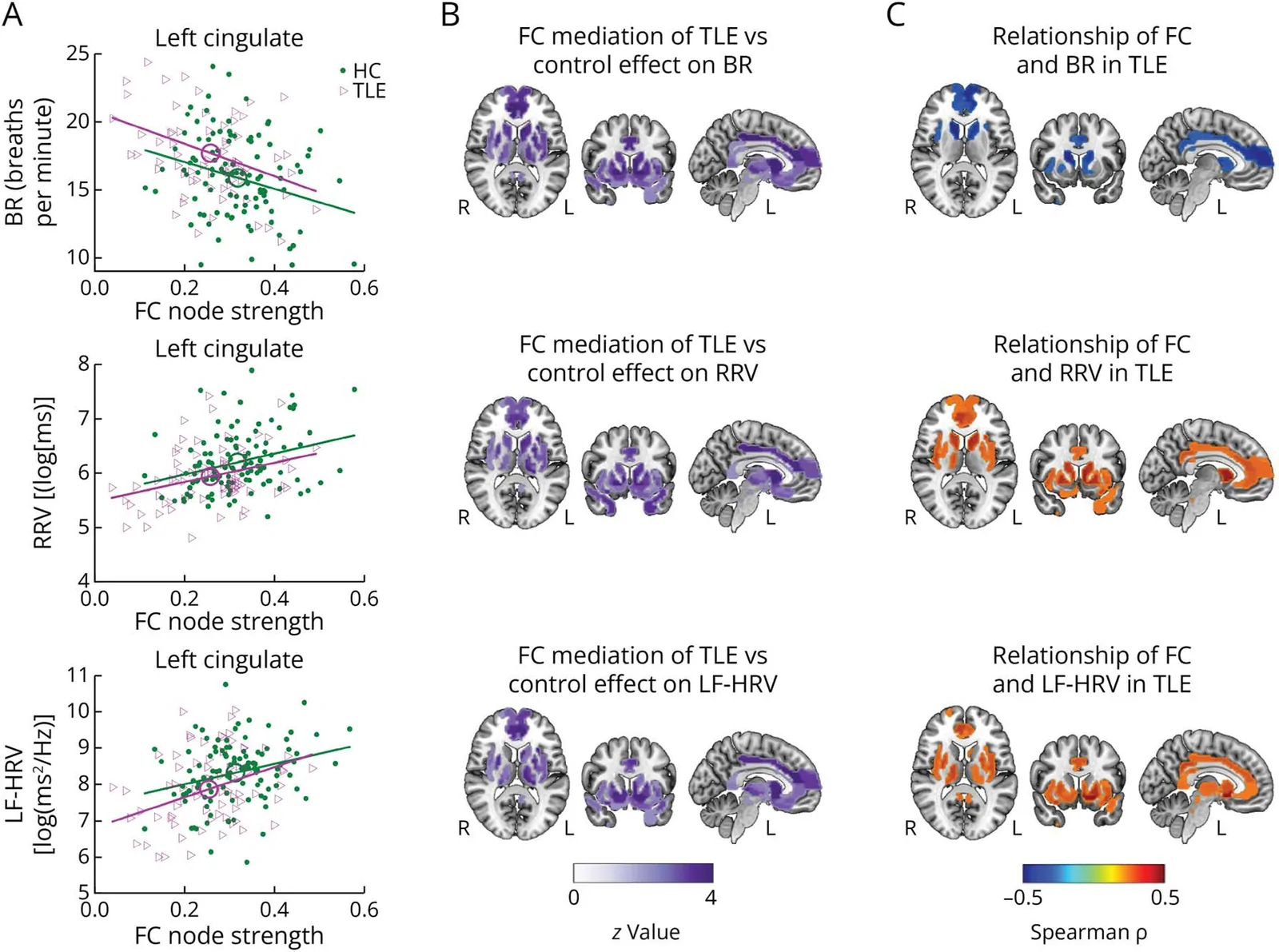
Reduced Functional Connectivity Strength Within the Central Autonomic Network Mediates Abnormal Interictal Cardiorespiratory Function in TLE (A) Relationship between the functional connectivity (FC) strength of a node in the left cingulate gyrus with breathing rate (BR), respiratory rate variability (RRV), and low-frequency heart rate variability (LF-HRV) in temporal lobe epilepsy (TLE) compared with healthy controls (HC). (B) Regional mediation effects of the FC node strength of the central autonomic network on differences in BR, RRV, and LF-HRV between TLE and controls (*p* < 0.05, false discovery rate [FDR]–corrected across regions; bootstrapping tests). The absolute values of the bootstrap *z*-scores are shown for ease of comparison. (C) Regional associations between the FC node strength of the central autonomic network with BR, RRV, and LF-HRV in TLE (*p* < 0.05, FDR-corrected across regions; 2-tailed Spearman correlation tests). The FC and cardiorespiratory estimates were adjusted for age and sex.

Moderation models indicated that reduced FC strength within the central autonomic network was associated with higher BR for 82 ROIs, lower RRV for 104 ROIs, and lower LF-HRV for 105 ROIs, independent of group effects (|Cohen’s *f*| = 0.16 to 0.52; *p*_FDR_ < 0.05; Figure 2A and eFigure 3). The patients and controls did not have a significant difference in the slope of the relationship between FC strength and cardiorespiratory measures for any ROIs after FDR correction. However, reduced FC strength in multiple nodes of the autonomic subnetwork mediated differences in cardiorespiratory function between TLE and controls (84 ROIs for BR, 77 ROIs for RRV, and 81 ROIs for LF-HRV; *p*_FDR_ < 0.05; Figure 2B and eTable 5).

We identified ROIs in which FC was both (1) reduced in TLE relative to controls and (2) related to cardiorespiratory measures in TLE (*p*_FDR_ < 0.05; Figure 2C and eTable 3). For BR, these regions included the fusiform gyrus, insula, cingulate, frontal pole, and basal ganglia. For RRV, these regions included the left hippocampus, parahippocampal gyrus, fusiform gyrus, insula, cingulate, right thalamus, right orbitofrontal cortex, frontal pole, basal ganglia, parabrachial nuclear complex (PBC), and left nucleus basalis of Meynert (NBM). For LF-HRV, these regions included the left hippocampus, left parahippocampal gyrus, fusiform gyrus, insula, cingulate, thalamus, orbitofrontal cortex, right frontal pole, basal ganglia, mesencephalic reticular formation (mRt), PBC, and left BNST.

### Clinical Correlates of Cardiorespiratory Function in TLE

GLMs revealed that seizure laterality had independent effects on RRV and LF-HRV (*p*_FDR_ < 0.05) but only nonsignificant effects on BR and HF-HRV (Table 2 and eFigure 4), with left-lateralized TLE exhibiting more abnormal cardiorespiratory function (higher BR, lower RRV, lower HF-HRV, and lower LF-HRV) compared with right-lateralized TLE. Although nonsignificant, higher BR was associated with higher seizure frequency and longer epilepsy duration, lower RRV was associated with later age at onset, and lower LF-HRV was associated with longer epilepsy duration and earlier age at onset. Similar results were obtained for GLMs fitted on matched left and right TLE subgroups (eTable 6). We also examined the relationship between the cardiorespiratory measures in TLE, finding that lower RRV was related to higher BR and lower HF-HRV and LF-HRV (*p*_FDR_ < 0.05; Table 2).

### Multivariate Associations of Brain Connectivity With Cardiorespiratory and Clinical Measures in TLE

**Figure 3:**
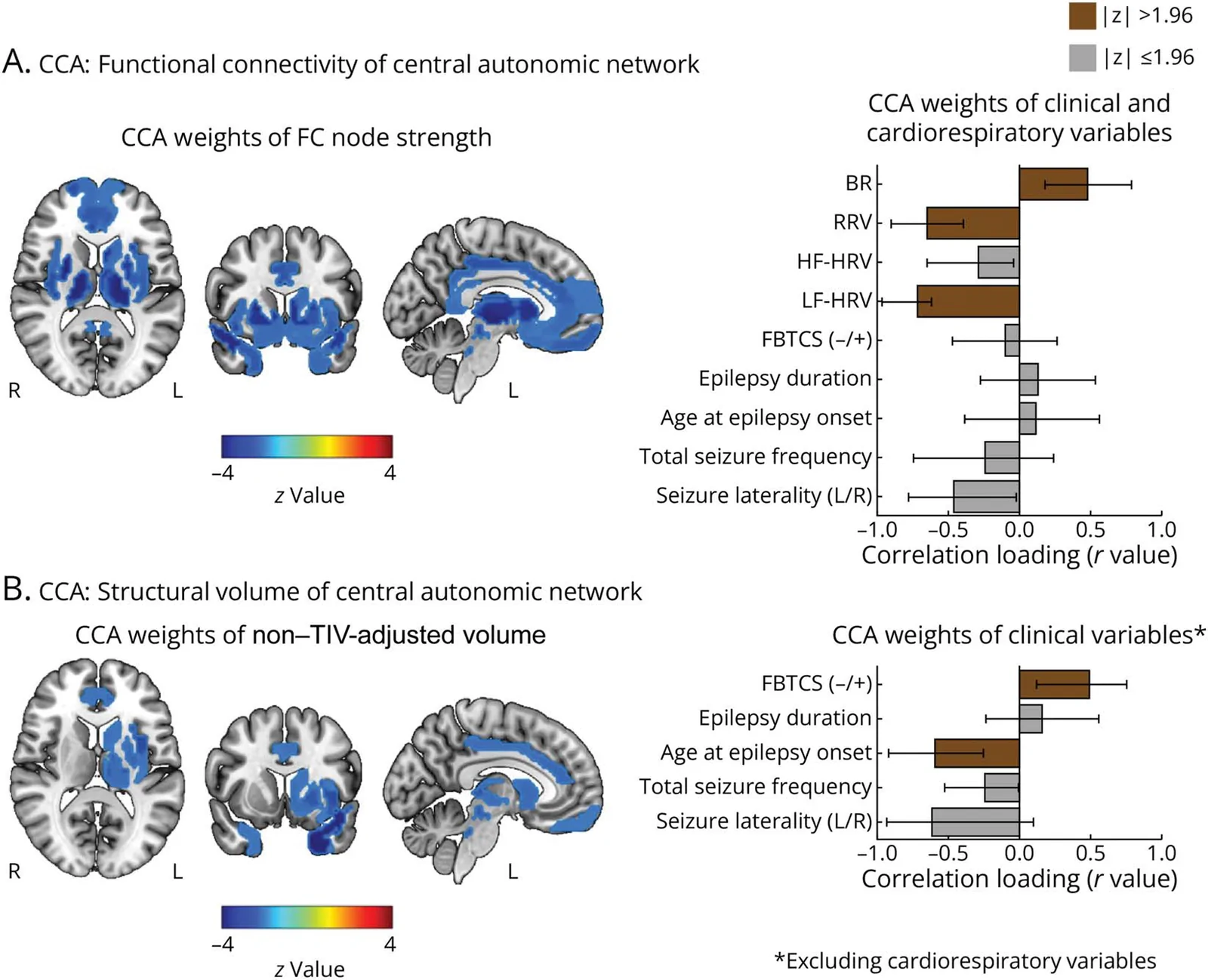
Multivariate Associations Between Clinical Disease Parameters, Interictal Cardiorespiratory Function, and the Central Autonomic Network in TLE (A) Loading weights of the canonical correlation analysis (CCA) mode that maximized the correlation between the functional connectivity (FC) strength of the central autonomic network and the combined set of cardiorespiratory measures and clinical parameters in temporal lobe epilepsy (*r* = 0.63; *p* = 0.015, familywise error rate [FWER]–corrected across 4 modes; permutation test). Contribution of the FC node strength of the central autonomic regions to the CCA mode (|*z*| > 1.96; bootstrap *z*-scores; *left brain plot*). Contribution of the cardiorespiratory measures and clinical parameters to the CCA mode (correlation loadings; *right bar plot*). (B) Loading weights of the CCA mode that maximized the correlation between the structural volume of the central autonomic network and the clinical parameters in temporal lobe epilepsy (*r* = 0.61; *p* = 0.018, FWER-corrected across 5 modes; permutation test). Contribution of the volume of the central autonomic regions to the CCA mode (|*z*| > 1.96; bootstrap z-scores; *left brain plot*). Contribution of the clinical parameters to the CCA mode (correlation loadings; *right bar plot*). Error bars in the bar plots correspond to 95% bootstrap CIs. The FC, cardiorespiratory, and brain volume estimates were adjusted for age and sex. The brain volume estimates were not adjusted for total intracranial volume (TIV). BR = breathing rate; FBTCS = focal-to-bilateral tonic-clonic seizures; HF-HRV = high-frequency HRV; LF-HRV = low-frequency heart rate variability; RRV = respiratory rate variability.

CCA identified 1 latent mode that maximized the correlation between the FC strength of the autonomic ROIs and the combined set of cardiorespiratory measures and clinical parameters in TLE (first latent mode out of 4; *r* = 0.63, *p*_FWER_ = 0.015; Figure 3A). The loading weights indicated that reduced FC strength (97 ROIs with *z* < −1.96) was strongly associated with lower RRV (*r* = −0.64, *z* = −2.75) and lower LF-HRV (*r* = −0.71, *z* = −3.48), moderately associated with higher BR (*r* = 0.48, *z* = 2.31), and nonsignificantly associated with left seizure laterality (*r* = −0.46, *z* = −1.87). Brain areas whose FC strength most reliably contributed to the CCA mode (*z* < −3) included the temporal lobe, insula, right cingulate, medial prefrontal cortex, thalamus, basal ganglia, and 4 brainstem nuclei (mRt, PBC, pontine oralis, and median raphe; eTable 7).

### Multivariate Associations of Brain Atrophy With Clinical Parameters of Disease Severity in TLE

The regional volume estimates were not significantly related to the cardiorespiratory measures after FDR correction. Similarly, none of the 5 CCA modes showed a significant correlation of the volume estimates with the set of cardiorespiratory measures and clinical parameters in TLE.

We then performed CCA while excluding the cardiorespiratory measures because they showed no association with brain volume. One latent mode was identified that maximized the correlation between the non–TIV-adjusted volume of the central autonomic network and the 5 clinical parameters (first latent mode out of 5; *r* = 0.61, *p*_FWER_ = 0.018; Figure 3B). The latent mode indicated that brain atrophy in TLE (60 ROIs with *z* < −1.96) was moderately associated with occurrence of FBTCS (*r* = 0.49, *z* = 2.37) and earlier age at onset (*r* = −0.59, *z* = −2.34) and nonsignificantly associated with left seizure laterality (*r* = −0.61, *z* = −1.68). Brain areas whose volume was most reliably related to the clinical parameters (*z* < −3) included the left temporal lobe, left insula, left thalamus, left NBM, and 6 brainstem nuclei (eTable 7). Similar results were obtained when performing CCA on TIV-adjusted volumes (eFigure 5).

### Impact of Postsurgical Changes in Autonomic Network Connectivity on Cardiorespiratory Function

**Figure 4:**
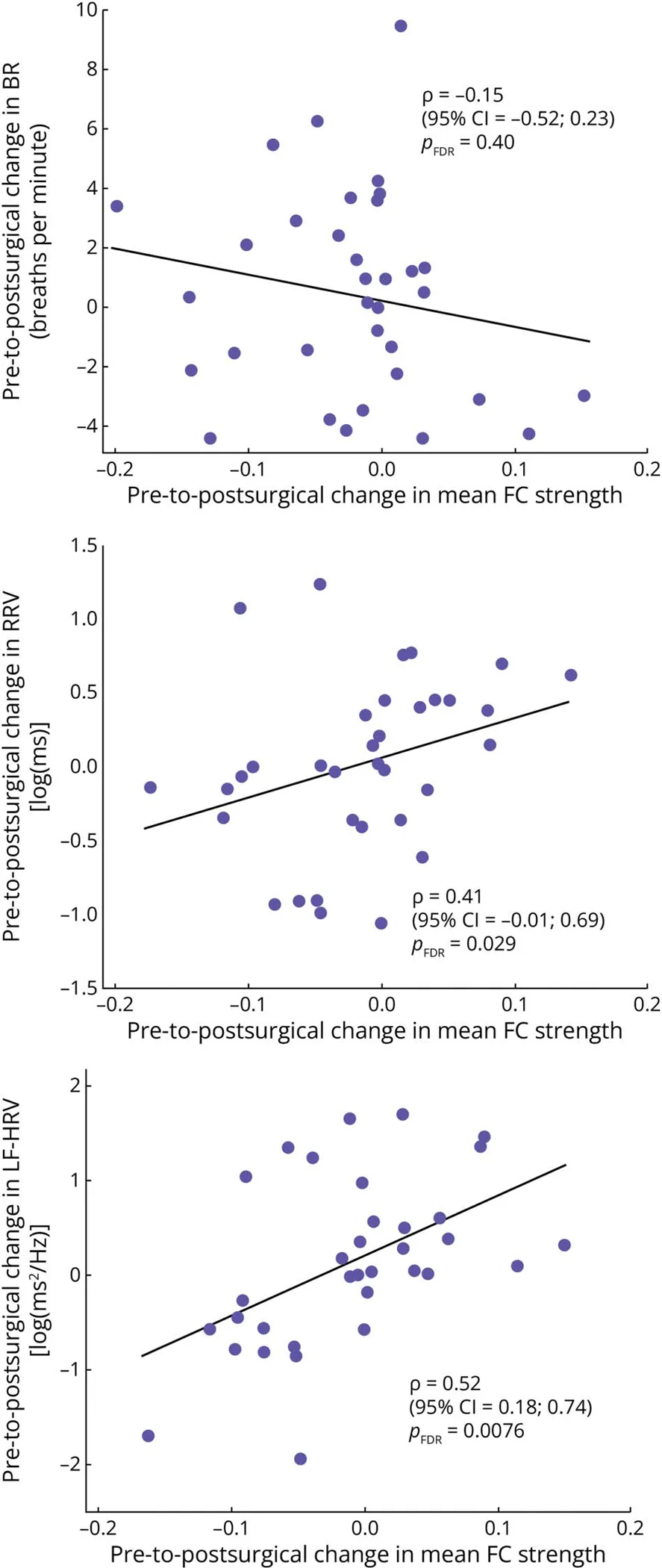
Postsurgical Changes in the Functional Connectivity Strength of the Central Autonomic Network Impact Cardiorespiratory Function in TLE Relationship of presurgical-to-postsurgical alterations in the mean functional connectivity (FC) strength of the central autonomic regions with presurgical-to-postsurgical changes in breathing rate (BR), respiratory rate variability (RRV), and low-frequency heart rate variability (LF-HRV). The FC and cardiorespiratory measures were adjusted for age and sex. Partial Spearman correlations (2-tailed) were used to test for a significant association between the FC and the cardiorespiratory measures while controlling for type of surgery (anterior temporal lobectomy or amygdalohippocampectomy) and time between surgery and postsurgical scan. Bootstrapping (n = 1 × 10^5^) was used to derive 95% CIs for the correlation values, and *p*-values were false discovery rate (FDR)–corrected across 3 cardiorespiratory measures. TLE = temporal lobe epilepsy.

We computed the presurgical and postsurgical FC node strength within the autonomic subnetwork for the patients after excluding temporal lobe regions. Epilepsy duration was greater for patients with postsurgical MRI compared with patients without postsurgical MRI, but there were no significant differences in cardiorespiratory measures or FC strength (eTable 8). Before surgery, reduced FC strength was related to higher BR for 20 ROIs, lower RRV for 43 ROIs, and lower LF-HRV for 49 ROIs (|ρ| = 0.26 to 0.49; *p*_FDR_ < 0.05; eFigure 6). After surgery, greater presurgical-to-postsurgical alterations in the mean FC of these regions were associated with greater changes in LF-HRV (ρ = 0.52, *p*_FDR_ = 0.0076; Figure 4 and eTable 9) and RRV (ρ = 0.41, *p*_FDR_ = 0.029) but nonsignificantly associated with BR (ρ = −0.15, *p*_FDR_ = 0.40). Corresponding results for the subgroup of patients who underwent selective amygdalohippocampectomy are presented in eTable 10. Increased LF-HRV after surgery was related to better seizure outcome (lower Engel score; ρ = −0.38, *p*_uncorrected_ = 0.033; eTable 11) and greater time after surgery (ρ = 0.40, *p*_uncorrected_ = 0.022).

## Discussion

The present study investigated the influence of long-term autonomic network alterations on cardiorespiratory function and higher risk of SUDEP in TLE. We demonstrated that patients with TLE exhibit decreased functional network strength and volume relative to healthy individuals in wide-spread central autonomic regions, including in multiple subcortical neuroregulatory nuclei. Reduced functional network strength mediated atypical respiratory rate and cardiac autonomic regulation in TLE, and we provided evidence that perturbation of autonomic network connectivity through surgery is associated with changes in cardiorespiratory function. These results indicate that seizure-related reorganization of neuroregulatory circuits may contribute to chronic autonomic and interoceptive deficits in TLE. Finally, we demonstrated that cardiorespiratory abnormalities were more prominent in left compared with right TLE and that atrophy in central autonomic regions was related to earlier age at epilepsy onset and the occurrence of generalized seizures, the strongest risk factor of SUDEP.^35^

We observed that LF-HRV was significantly decreased and HF-HRV was nonsignificantly decreased in TLE relative to controls. These results agree with prior literature showing that patients with TLE present with interictal reductions in baroreflex sensitivity, LF-HRV, and HF-HRV.^3,5^ Reduced LF-HRV may reflect chronic dysfunction in both sympathetic and parasympathetic regulation as well as the baroreflex.^26^ Previous studies have reported that interictal normalized LF-HRV and time-domain HF-HRV were lower in SUDEP cases compared with epilepsy controls.^7,28^ In our cohort, longer epilepsy duration and earlier age at onset were only non-significantly associated with decreased LF-HRV. Therefore, further work is needed to determine the relationship between interictal autonomic dysfunction and SUDEP risk. We also found that reduced LF-HRV was related to a greater number of AEDs (ρ = −0.28, *p*_uncorrected_ = 0.017; eTable 12). This may be relevant to SUDEP pathophysiology considering that AED polytherapy is a SUDEP risk factor.^35^ However, it remains unclear whether the effects of polytherapy represent more severe epilepsy or influence of certain AED regimens on autonomic function.^3^

In addition to altered HRV, patients with TLE had higher BR and lower RRV compared with controls, suggesting chronic respiratory dysfunction. Few studies to date have investigated interictal respiratory function in human epilepsy. One group demonstrated that the hypercapnic response was impaired in patients with epilepsy and related to higher interictal RRV and more severe respiratory depression following TCS.^4,8,29^ Animal studies have reported similar results. For instance, mouse models of SUDEP show higher interictal BR and RRV relative to wild-type mice.^30^ Furthermore, our findings indicate that abnormal RRV may contribute to lower HRV in epilepsy, possibly reflecting a common source of cardiorespiratory dysfunction.

A limitation of our work is that cardiorespiratory function measured during fMRI may not generalize to naturalistic environments or more controlled experimental conditions. Particularly, a nonsignificant finding for reduced HF-HRV in TLE may be due to differences in alertness.^26^ Sensitivity analyses indicated that cardiorespiratory measures had excellent reliability between fMRI scans (eTable 13). However, there was a systematic increase in HRV during the second scan that may be attributed to reduced alertness. Some evidence indicates that patients experience greater drowsiness during fMRI than controls, although additional investigation using vigilance measures (such as EEG) is needed.^17^ Notably, effect sizes for BR, RRV, and LF-HRV differences between TLE and controls were comparable with HRV alterations reported in SUDEP cases,^7,28^ suggesting that these measures may be clinically relevant for SUDEP risk prediction.

Our results suggest that patients with TLE exhibit widespread FC reductions in the central autonomic network relative to healthy individuals, including in the thalamus, hypothalamus, NBM, BNST, and 5 brainstem arousal nuclei. These findings align with our previous studies showing that patients with TLE have abnormally decreased connectivity in neuroregulatory subcortical-to-cortical networks.^16,17^ This study and previous work also found atrophy of neuroregulatory centers in TLE that was related to greater disease severity and the occurrence of generalized seizures,^14,39^ providing further evidence of chronic disruption.

We demonstrated that reduced autonomic network strength mediated abnormal cardiorespiratory function and that the connectivity of multiple neuroregulatory centers was associated with RRV and LF-HRV in TLE. These nuclei included the PBC, mRt, left BNST, left NBM, and prefrontal thalamus, which are involved in arousal and cardiorespiratory regulation. In particular, the PBC is a critical modulator of respiratory phase switching, arousal responses to hypercapnia, and autonomic control of blood pressure.^24^ The BNST is an extended amygdalar structure that relays information between cortical autonomic regions and brainstem cardiorespiratory centers, and previous studies suggest that BNST projections to the PBC have reduced neural excitability in mouse models of Dravet syndrome and SUDEP.^10,40^ Our results also revealed that the connectivity of the median raphe was associated with interictal cardiorespiratory function, although we did not find a significant difference in its connectivity between TLE and controls. We implemented preprocessing strategies to mitigate nonneural noise, but conventional 3T fMRI may still be limited by signal-to-noise ratio and spatial precision challenges in the brainstem.^18^

Beyond these neuroregulatory centers, reduced functional network strength in the basal ganglia, hippocampus, temporal cortex, medial prefrontal cortex, insula, and cingulate was related to interictal cardiorespiratory dysfunction in TLE. Previous studies in healthy participants have shown that FC of autonomic regions is positively correlated with HRV,^41^ consistent with our findings. In addition, hypercapnia and air hunger lead to activations in a widespread network including the brainstem, hypothalamus, basal ganglia, amygdala, parahippocampal gyrus, fusiform gyrus, insula, and cingulate.^23^ Long-term connectivity alterations in these regions may contribute to impaired respiratory interoception during seizures.^11^ A recent study also indicates that dynamic FC states of the insula might be promising biomarkers of SUDEP risk.^42^

Our primary hypothesis is that abnormally decreased connectivity in TLE represents disrupted neural communication within the central autonomic network. However, alterations in breathing and heart rate are associated with global non-neural BOLD changes,^18^ which may contribute to widespread connectivity differences between TLE and controls. We regressed out tissue-based confound signals to mitigate non-neural effects.^18^ Moreover, patients with TLE have wide-spread abnormalities in brain structure and electrophysiologic phase synchrony, indicating a neural basis of global network dysfunction.^14,15,34^

CCA linked atrophy in the temporal lobe, insula, thalamus, NBM, and brainstem arousal centers to the occurrence of FBTCS in TLE. Similarly, previous work suggests that thalamic and brainstem volume loss is a biomarker of TCS and elevated SUDEP risk.^39,43^ However, higher SUDEP risk has also been associated with volume gain in mesial temporal structures,^43^ which is inconsistent with our findings. Discrepancies may arise from differences in study population considering that we only analyzed unilateral TLE. Additionally, we did not examine relationships with structural lesions or peri-ictal cardiorespiratory abnormalities.

A notable finding was that left seizure laterality was independently related to abnormal cardiorespiratory function in TLE. One study reported that LF-HRV was lower in left compared with right TLE,^44^ consistent with our results. Some evidence suggests that sympathetic and parasympathetic activity is predominately modulated by the right and left hemispheres, respectively.^9^ Therefore, abnormal HRV in TLE may represent greater dysfunction in parasympathetic than sympathetic regulation. Another explanation for seizure laterality effects on interictal cardiorespiratory function may be that the left hemisphere is especially vulnerable to lasting injury.^45^ Previous studies have shown that left TLE is associated with greater structural abnormalities than right TLE.^14,45^ Although the relationship was nonsignificant, CCA indicated that left seizure laterality might be associated with greater atrophy and FC reductions in the central autonomic network. The disease variables in CCA also exhibited mostly left-lateralized effects on brain volume.

The effects of epilepsy surgery on interictal cardiorespiratory function have been investigated. One group reported that baroreflex sensitivity and LF power of heart rate were reduced following temporal lobectomy in TLE,^46^ while others reported no presurgical-to-postsurgical differences in LF-HRV.^47^ Although we did not find a significant difference in cardiorespiratory measures from presurgery to postsurgery, our results suggest that perturbation of autonomic network connectivity toward or away from control levels may explain why some patients experience normalization or worsening of cardiorespiratory function after surgery. A discrepancy with previous work is that most of our cohort underwent selective amygdalohippocampectomy. We did not find an effect of surgery type on changes in FC or cardiorespiratory function (eTables 9 and 11). This may be due to insufficient statistical power considering that larger TLE resections have been associated with greater changes in brain structure.^48^

In patients whom resection surgery is contraindicated or seizure freedom cannot be obtained, novel neuromodulation therapies may restore cardiorespiratory function. Our results implicate abnormal connectivity of the prefrontal thalamus, NBM, and BNST in interictal cardiorespiratory dysfunction. Neurostimulation of intralaminar thalamic nuclei is being explored for treatment of consciousness-impairing seizures.^49^ The NBM has been proposed as another feasible target for arousal restoration in epilepsy,^16^ and neuromodulation of the BNST has been established as a safe treatment for obsessive compulsive disorder.^50^ Neurostimulation of the NBM and thalamus may improve cardiorespiratory function because of the overlapping neural circuitry between the arousal system and central autonomic network.^24^ However, future studies replicating our findings in external cohorts, examining SUDEP cases, and relating interictal to ictal autonomic network dysfunction are needed to fully understand the role of these brain targets in SUDEP pathophysiology. We also caution that our study provides correlational rather than causal evidence of a mechanistic link between autonomic network disruptions and cardiorespiratory dysfunction in TLE.

## Supplementary Material

Supplementary

## Figures and Tables

**Table 1:** Participant Demographics and Clinical Information

	Controls (n = 105)	Full TLE cohort (n = 72)	Postsurgical TLE subcohort (n = 34)
**Sex (M/F), n**	49/56	37/35^a^	19/15
**Age at presurgical scan (y), mean ± SD**	36.7 ± 12.8	40.0 ± 12.8^b^	42.6 ± 11.8
**MRI scanner version at presurgical scan (version 1/version 2), n**	37/68	20/52^c^	9/25
**FBTCS occurrence (no/yes/unknown), n**	—	30/40/2	15/18/1
**Epilepsy duration at presurgical scan (y), mean ± SD**	—	18.3 ± 14.2	23.4 ± 16.3
**Age at epilepsy onset (y), mean ± SD**	—	21.7 ± 16.3	19.2 ± 14.6
**Total seizure frequency (per year), mean ± SD**	—	16.3 ± 36.7	8.4 ± 8.0
**Seizure laterality (left/right), n**	—	31/41	14/20
**No. of antiepileptic drugs, median (IQR)**	—	2 (2–3)	2 (2–2)
**MTS on MRI (no/yes), n** ^d^	—	33/39	12/22
**Surgery type, n**			
**SAH**		46	25
**TL**		14	8
**SLAH**		3	1
**Mesial temporal RNS**		5	0
**Temporal encephalocele lesionectomy**		1	0
**None**		3	0
**Time between presurgical and postsurgical scans (mo), mean ± SD**	—	—	28.9 ± 16.3
**Time between surgery and postsurgical scan (mo), mean ± SD**	—	—	26.6 ± 16.5
**Engel outcome at postsurgical scan (IA/IB-D/II/III/IV), n**	—	—	17/6/4/6/1

Abbreviations: FBTCS = focal-to-bilateral tonic-clonic seizures; IQR = interquartile range; MTS = mesial temporal sclerosis; RNS = responsive neurostimulation; SAH = selective amygdalohippocampectomy; SLAH = stereotactic laser amygdalohippocampectomy; TL = temporal lobectomy; TLE = temporal lobe epilepsy.

a *p* = 0.54, uncorrected; full TLE cohort vs controls; Fisher exact test.

b *p* = 0.093, uncorrected; full TLE cohort vs controls; two-sample *t* test.

c *p* = 0.33, uncorrected; full TLE cohort vs controls; Fisher exact test.

d Other lesions on MRI included temporal encephalocele for the full TLE cohort (n = 5) and postsurgical TLE subcohort (n = 1).

**Table 2:** Associations Between Cardiorespiratory Measures and Clinical Parameters in TLE

Multivariate models (multiple linear regression)^a^								

	BR		RRV		HF-HRV		LF-HRV	
	Cohen *f*	*p*_FDR_	Cohen *f*	*p*_FDR_	Cohen *f*	*p*_FDR_	Cohen f	*p*_FDR_

**FBTCS occurrence (yes, ref: no)**	—	—	—	—	—	—	—	—

**Epilepsy duration**	0.21	0.17	—	—	—	—	−0.073	0.55

**Age at epilepsy onset**	—	—	−0.15	0.28	—	—	0.11	0.41

**Total seizure frequency**	0.17	0.24	—	—	—	—	—	—

**Seizure laterality (left, ref: right)**	0.25	0.097	−0.45^b^	0.0046^b^	−0.28	0.065	−0.42^b^	0.0055^b^

Pairwise analyses (Spearman correlation test)								

	BR		RRV		HF-HRV		LF-HRV	
	ρ	*p*_FDR_	ρ	*p*_FDR_	ρ	*p*_FDR_	ρ	*p*_FDR_

**BR**	—	—	−0.59^b^	<0.001^b^	−0.13	0.29	−0.2	0.12

**RRV**	−0.59^b^	<0.001 ^b^	—	—	0.3^b^	0.017^b^	0.43^b^	<0.001 ^b^

**HF-HRV**	−0.13	0.29	0.3^b^	0.017^b^	—	—	0.82^b^	<0.001 ^b^

**LF-HRV**	−0.2	0.12	0.43^b^	<0.001^b^	0.82^b^	<0.001 ^b^	—	—

Abbreviations: BR = breathing rate; FBTCS = focal-to-bilateral tonic-clonic seizures; HF-HRV = high-frequency heart rate variability; LF-HRV = low-frequency heart rate variability; RRV = respiratory rate variability; TLE = temporal lobe epilepsy.

a A rank-based inverse normal transformation was applied to ensure normality of the residuals, and Least Absolute Shrinkage and Selection Operator regression with leave-one-out cross-validation was used for variable selection. The effect size (Cohen’s *f*) is weighted by the sign of the beta coefficient to indicate directionality.

b *p* < 0.05, false discovery rate (FDR)–corrected across cardiorespiratory measures and clinical parameters.

## Glossary

AED: antiepileptic drug
BNST: bed nucleus of the stria terminalis
BR: breathing rate
CCA: canonical correlation analysis
FBTCS: focal-to-bilateral tonic-clonic seizures
FC: functional connectivity
FDR: false discovery rate
FWER: familywise error rate
GLM: general linear model
HF-HRV: high-frequency heart rate variability
HRV: heart rate variability
LF-HRV: low-frequency heart rate variability
mRt: mesencephalic reticular formation
NBM: nucleus basalis of Meynert
PBC: parabrachial nuclear complex
PPG: photoplethysmography
ROI: region of interest
RRV: respiratory rate variability
SUDEP: sudden unexpected death in epilepsy
TCS: tonic-clonic seizure
TIV: total intracranial volume
TLE: temporal lobe epilepsy
