# Supplementary material for: Autonomic Network Signatures of Interictal Cardiorespiratory Dysfunction in Patients With Temporal Lobe Epilepsy
Source: Neurology. Author manuscript; Available in PMC 2026 Sep 24. (PMC13552367; doi:10.1212/WNL.0000000000218470)
Supplement: Supplementary file 1 [file NIHMS2209141-supplement-Supplementary.pdf]

Supplemental Material

eMethods 1
eFigure 1. 4
eTable 1. 5
eTable 2. 5
eFigure 2. 6
eFigure 3. 7
eFigure 4. 8
eFigure 5. 9
eTable 3. 10
eTable 4. 11
eTable 5. 12
eTable 6. 13
eTable 7. 14
eTable 8. 15
eFigure 6. 16
eTable 9. 17
eTable 10. 17
eTable 11. 18
eTable 12. 19
eTable 13. 19
eReferences 20

eMethods

MRI acquisition parameters

The MRI data were acquired on Philips 3T MRI scanners (Philips Healthcare, Best, Netherlands) using a 32-channel head coil.[1,2] Each session consisted of two consecutive 10-min scans of BOLD fMRI collected in an eyes-closed, resting-state condition (echo planar imaging; 3×3-mm in-plane resolution; 80×80 matrix; 3.5-mm slice thickness; 0.5-mm slice gap; 34 axial slices; TR = 2000 ms; TE = 35 ms; FA = 79°; N = 300 volumes). A T1-weighted axial 2D MRI scan was acquired for functional to structural co-registration (turbo spin echo; 1×1-mm in-plane resolution; 256×256 matrix; 3.5-mm slice thickness; 0.5-mm slice gap; 34 axial slices; TR = 2000 ms; TE = 10 ms; TI = 800 ms; FA = 90°; TSE factor = 7), and a T1-weighted 3D MRI scan was obtained for spatial normalization and brain volume estimation (turbo field echo; 1×1×1-mm resolution; 256×256 matrix; 150 axial slices; TR = 9.1 ms; TE = 4.6 ms; FA = 8°; TFE factor = 128). Respiratory and photoplethysmography (PPG) data were recorded at 500 or 496 Hz using a scanner-integrated pneumatic belt and PPG transducer.

fMRI preprocessing steps

The fMRI data were preprocessed with RETROICOR to remove non-BOLD physiological artifacts.[3] SPM12 was then used to perform slice-timing correction, motion co-registration, and normalization to the MNI152 template (3-mm isotropic resolution). Additional preprocessing included regression of nuisance signals followed by bandpass filtering at 0.0067-0.15 Hz. The nuisance signals consisted of six motion realignment parameters and their first-order temporal derivatives; mean white matter, deep CSF, and fourth ventricle signals; and up to fourth order polynomials (to remove scanner drift). RETROICOR and the nuisance regression were performed to improve the signal-to-noise ratio in the brainstem, basal forebrain, and hypothalamus.[4,5]

Mean fMRI signals were extracted from regions of interest (ROIs) resampled to MNI space at 3-mm isotropic resolution. To avoid loss of spatial resolution, spatial smoothing was not applied to the fMRI data.[5] To assess possible spatial overlap at the fMRI acquisition resolution, masks of the 10 brainstem regions of Harvard Ascending Arousal Network atlas were visualized at 3×3×4 mm (**eFigure 1**). Dice similarity coefficients were computed between brainstem masks to quantify the degree of spatial overlap, and we confirmed that there was no spatial overlap between ROIs (Dice coefficient = 0 for all ROI pairs).

Computation of cardiac and respiratory measures

Heart rate variability (HRV) was calculated based on the inter-beat interval derived from the PPG data.[6] Outliers in the inter-beat interval were linearly interpolated. The Lomb-Scargle method was then employed to estimate the power spectrum of the inter-beat interval in 60-s windows (50% overlap), and LF and HF-HRV were computed by taking the logarithm of the power spectrum averaged over 0.04-0.15 and 0.15-0.4 Hz.[6,7] The HRV metrics were averaged across all windows per participant.

Breathing rate (BR) and respiratory rate variability (RRV) were calculated based on the inter-breath interval derived from the respiratory recordings.[8] A custom algorithm was used to identify and exclude sensor-related dropout periods, and outliers in the inter-breath interval were linearly interpolated. BR was then computed by taking the inverse of the mean inter-breath interval within 60-s windows (50% overlap), and RRV was computed by taking the logarithm of the SD of the inter-breath interval.[9] The BR and RRV were averaged across all windows per participant.

Matched analysis of seizure laterality effects on cardiorespiratory function

The results of the main GLM analysis indicated that left-lateralized TLE exhibited lower RRV and LF-HRV compared to right-lateralized TLE, independent of clinical parameters of disease severity. To evaluate the robustness of these effects to potential imbalances between the right and left TLE subgroups, we repeated the GLM analysis after matching patients in the two subgroups based on propensity scores derived from the nine demographic and clinical variables listed in **eTable 1**. Linear propensity scores were estimated from a logistic regression model, and patients were optimally matched by minimizing the sum of all the propensity scores.[10] Consistent with the main GLM results, left-lateralized TLE was associated with lower RRV and LF-HRV ($p_{FDR} < 0.05$; **eTable 6**). Additionally, the matched analysis showed that left seizure laterality was related to lower HF-HRV ($p_{FDR} < 0.05$), whereas this effect was non-significant in the main GLM analysis after FDR correction.

Comparisons between patients with and without postsurgical MRI

The subcohort of patients with postsurgical MRI had greater epilepsy duration than patients without postsurgical MRI (**eTable 2**). To examine the generalizability across the two subgroups, we employed Wilcoxon rank-sum tests to compare the cardiorespiratory measures and extratemporal autonomic FC strength between patients with and without postsurgical MRI. None of the cardiorespiratory measures were significantly different between the patient subgroups (**eTable 8**). Compared to patients without postsurgical MRI, patients with postsurgical MRI had greater FC strength in nodes of the right orbitofrontal gyrus and frontal pole (two ROIs; $r = 0.27, 0.32$; $p_{uncorr} = 0.0062, 0.0244$). These differences in FC did not survive FDR correction across ROIs.

Postsurgical changes in patients with selective amygdalohippocampectomy

In the postsurgical subcohort ($n = 34$), pre-to-postsurgical alterations in mean autonomic FC strength were positively correlated with changes in LF-HRV and RRV but only non-significantly associated with BR (**eTable 9**). We evaluated the robustness of these associations to differences in surgery type by restricting the partial Spearman correlation analysis to patients who underwent selective amygdalohippocampectomy ($n = 25$). We found that effect sizes for associations between change in FC and cardiorespiratory measures were similar to the analysis on the entire postsurgical subcohort: LF-HRV ($rho = 0.46$ vs. 0.52; **eTable 10**), RRV ($rho = 0.35$ vs. 0.41), and BR ($rho = -0.20$ vs. -0.15). However, only the association with LF-HRV reached statistical significance at an uncorrected threshold in the restricted analysis ($p_{uncorr} = 0.025$), and this effect did not survive FDR correction across three cardiorespiratory measures ($p_{FDR} = 0.076$). This smaller effect may be driven primarily by loss of statistical power rather than a difference in surgery type, as there was no significant effect of surgery type in the analysis on the entire postsurgical subcohort.

Effects of antiepileptic drugs and mesial temporal sclerosis

We investigated the effects of two clinical variables (number of antiepileptic drugs [AEDs] and mesial temporal sclerosis [MTS]) on cardiorespiratory function and autonomic FC strength in patients with TLE. Spearman's correlation tests were used to evaluate associations with number of AEDs per patient, and Wilcoxon rank-sum tests were used to compare each cardiorespiratory and FC variable between patients with and without MRI evidence of MTS. GLMs were then employed to test for a moderation effect of each clinical variable on the relationship between FC and cardiorespiratory measures. Before the statistical analyses, cardiorespiratory and FC variables were adjusted for age, sex, and MRI scanner.

Before false discovery rate (FDR) correction, a greater number of AEDs was associated with lower LF-HRV ($p_{uncorr} = 0.017$; **eTable 12**) but only non-significantly associated with lower HF-HRV ($p_{uncorr} = 0.051$). The relationship with LF-HRV did not survive FDR correction across four cardiorespiratory measures. MRI evidence of MTS was not significantly associated with any cardiorespiratory measure.

Greater number of AEDs was related to reduced FC strength in nodes of the thalamus, left basal ganglia, and left BNST (four ROIs; *rho* = -0.30 to -0.23; p_{uncorr} = 0.010 to 0.049). Compared to patients without MTS, patients with MTS had greater FC strength in the PTg ($r = 0.26$; $p_{uncorr} = 0.025$) and reduced FC in nodes of the left insula (two ROIs; r = -0.30, -0.25; p_{uncorr} = 0.011, 0.034). None of these associations survived FDR correction across ROIs.

Number of AEDs moderated the relationship between FC and BR in two ROIs (Cohen's f = -0.36, -0.25; p_{uncorr} = 0.0041, 0.042), between FC and RRV in one ROI (Cohen's $f = 0.25$; $p_{uncorr} = 0.045$), and between FC and LF-HRV in four ROIs (Cohen's f = 0.25 to 0.38; p_{uncorr} = 0.0027 to 0.040). In addition, MRI evidence of MTS moderated the relationship between FC and BR in two ROIs (Cohen's f = -0.26, -0.27; p_{uncorr} = 0.035, 0.030), between FC and RRV in 13 ROIs (Cohen's f = 0.24 to 0.33; p_{uncorr} = 0.0080 to 0.050), between FC and HF-HRV in two ROIs (Cohen's f = -0.25, 0.28, p_{uncorr} = 0.044, 0.024), and between FC and LF-HRV in one ROI (Cohen's $f = 0.36$; $p_{uncorr} = 0.0041$). None of the moderation effects survived FDR correction across ROIs.

Reliability of cardiorespiratory measures

We used the intra-class correlation coefficient (ICC) to evaluate the test-retest reliability of each cardiorespiratory measure across the two fMRI scans per participant. The ICC(3,1) was computed from two-way analysis of variance (ANOVA) models.[11] To quantify systematic bias across scans, we also computed Cohen's d for difference scores of each cardiorespiratory measure between scans.

The reliability of all the cardiorespiratory measures was excellent (ICC $\geq$ 0.75) for both controls and patients with TLE (**eTable 13**). HF and LF-HRV were increased during the second relative to the first scan in both controls and patients (Cohen's d = 0.28 to 0.43). This systematic increase in HRV may result from greater drowsiness during the second scan.[12,13] However, the fMRI sessions did not include validated measures of vigilance (such as EEG) to confirm this hypothesis.

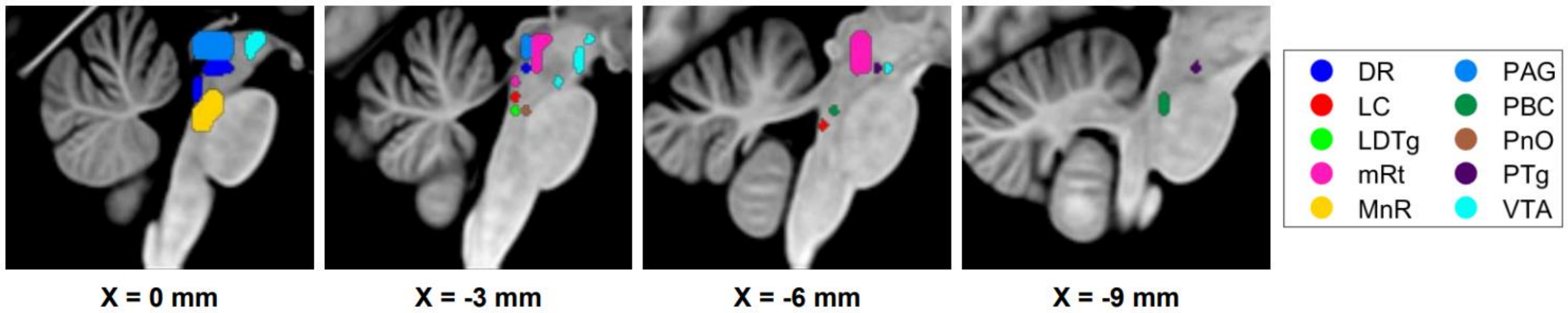

eFigure 1. Masks of the 10 brainstem regions from the Harvard Ascending Arousal Network atlas, spatially downsampled to the acquisition resolution of the fMRI data (3x3x4 mm). DR = dorsal raphe; LC = locus coeruleus; LDTg = laterodorsal tegmental nucleus; mRt = mesencephalic reticular formation; MnR = median raphe; PAG = periaqueductal gray; PBC = parabrachial nuclear complex; PnO = pontine oralis; PTg = pedunculotegmental nucleus; VTA = ventral tegmental area.

eTable 1. Comparison of demographic and clinical information between patients with left versus right TLE.

	**Left TLE (*n* = 31)**	**Right TLE (*n* = 41)**	***P* uncorr.[a]**
Sex (*n*, M/F)	20/11	17/24	0.061
Age at presurgical scan (years, mean ± SD)	40.4 ± 13.8	39.8 ± 12.1	0.85
MRI scanner version at presurgical scan (*n*, version 1/version 2)	12/19	8/33	0.11
FBTCS occurrence (*n*, no/yes/unknown)	10/21/0	20/19/2	0.15
Epilepsy duration at presurgical scan (years, mean ± SD)	18.2 ± 13.8	18.4 ± 14.6	0.94
Age at epilepsy onset (years, mean ± SD)	22.1 ± 17.4	21.3 ± 15.6	0.96
Total seizure frequency (per year, mean ± SD)	12.4 ± 16.8	19.2 ± 46.4	0.95
Number of antiepileptic drugs (median [IQR])	2 [2–2.75]	2 [2–3]	0.79
MTS on MRI (*n*, no/yes)	11/20	22/19	0.16

TLE = temporal lobe epilepsy; FBTCS = focal-to-bilateral tonic-clonic seizures; MTS = mesial temporal sclerosis.

a) Left versus right TLE; Fischer's exact tests for sex, MRI scanner version, FBTCS occurrence, and MTS on MRI; two-sample *t*-test for age at presurgical scan; Wilcoxon rank-sum tests for epilepsy duration, age at epilepsy onset, total seizure frequency, and number of antiepileptic medications.

eTable 2. Comparison of demographic and clinical information between patients with and without postsurgical MRI.

	**Non-postsurgical subcohort (*n* = 38)**	**Postsurgical subcohort (*n* = 34)**	***P* uncorr.[a]**
Sex (*n*, M/F)	18/20	19/15	0.49
Age at presurgical scan (years, mean ± SD)	37.8 ± 13.3	42.6 ± 11.8	0.11
MRI scanner version at presurgical scan (*n*, version 1/version 2)	11/27	9/25	1.00
FBTCS occurrence (*n*, no/yes/unknown)	15/22/1	15/18/1	0.81
Epilepsy duration at presurgical scan (years, mean ± SD)	**13.8 ± 10.3**	**23.4 ± 16.3**	**0.021**
Age at epilepsy onset (years, mean ± SD)	23.8 ± 17.5	19.2 ± 14.6	0.34
Total seizure frequency (per year, mean ± SD)	23.3 ± 49.1	8.4 ± 8.0	0.51
Seizure laterality (*n*, left/right)	17/21	14/20	0.81
Number of antiepileptic drugs (median [IQR])	2 [2–3]	2 [2–2]	0.35
MTS on MRI (*n*, no/yes)[d]	21/17	12/22	0.10

FBTCS = focal-to-bilateral tonic-clonic seizures; MTS = mesial temporal sclerosis.

Bold entries denotes statistically significant differences between patient subcohorts at $p < 0.05$.

a) Non-postsurgical versus postsurgical subcohort; Fischer's exact tests for sex, MRI scanner version, FBTCS occurrence, and MTS on MRI; two-sample *t*-test for age at presurgical scan; Wilcoxon rank-sum tests for epilepsy duration, age at epilepsy onset, total seizure frequency, and number of antiepileptic medications.

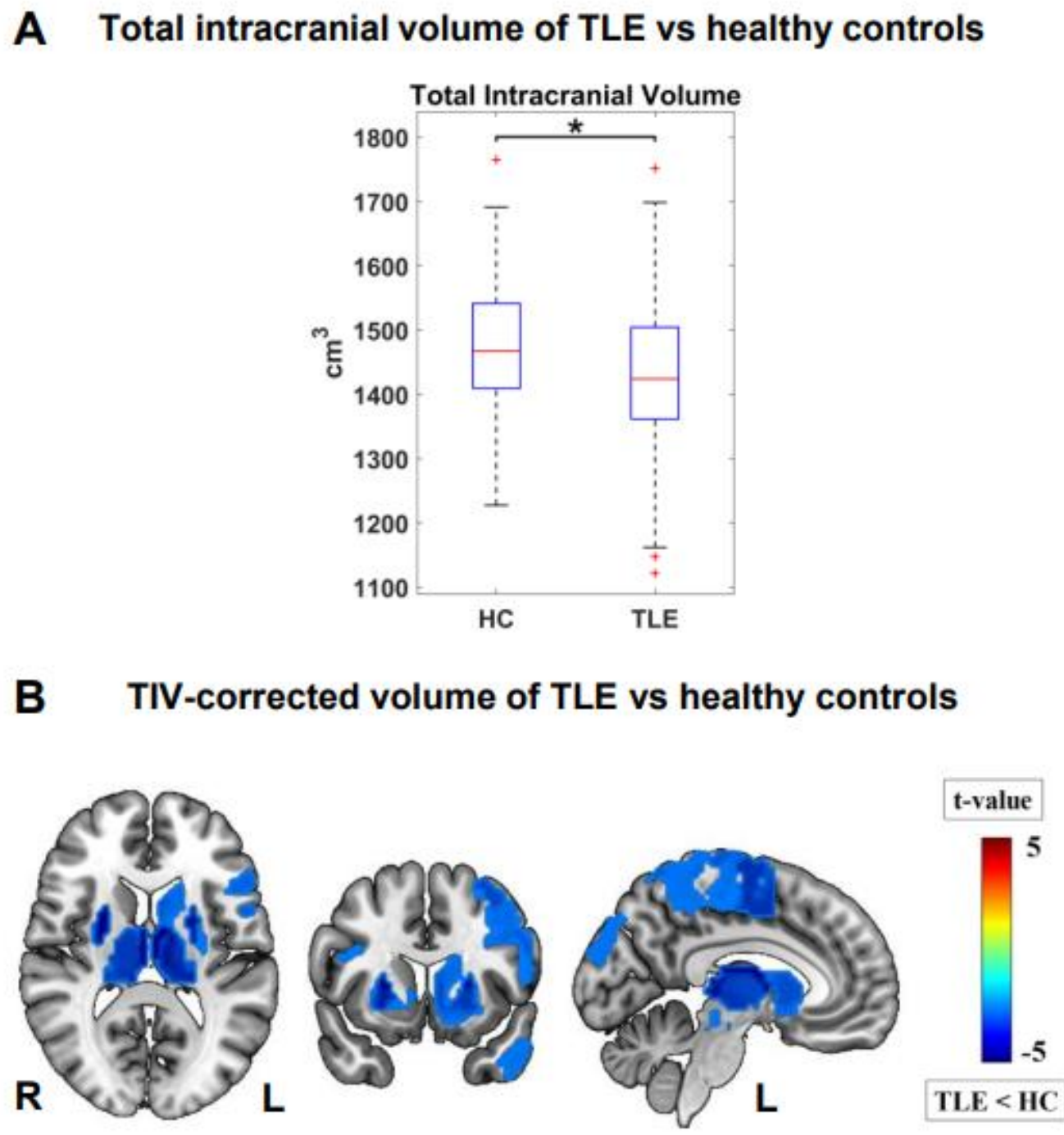

eFigure 2. (A) Comparison of total intracranial volume (TIV) between temporal lobe epilepsy (TLE) and healthy controls (HC). Two-samples *t*-tests (two-tailed) were used for statistical comparisons. $*p < 0.05$, $**p < 0.01$, $***p < 0.001$. The TIV estimates were adjusted for age and sex. **(B)** Regional differences in structural volume between TLE and controls while adjusting for age, sex, and TIV ($p < 0.05$, false discovery rate [FDR]-corrected across regions; two-sample *t*-tests, two-tailed).

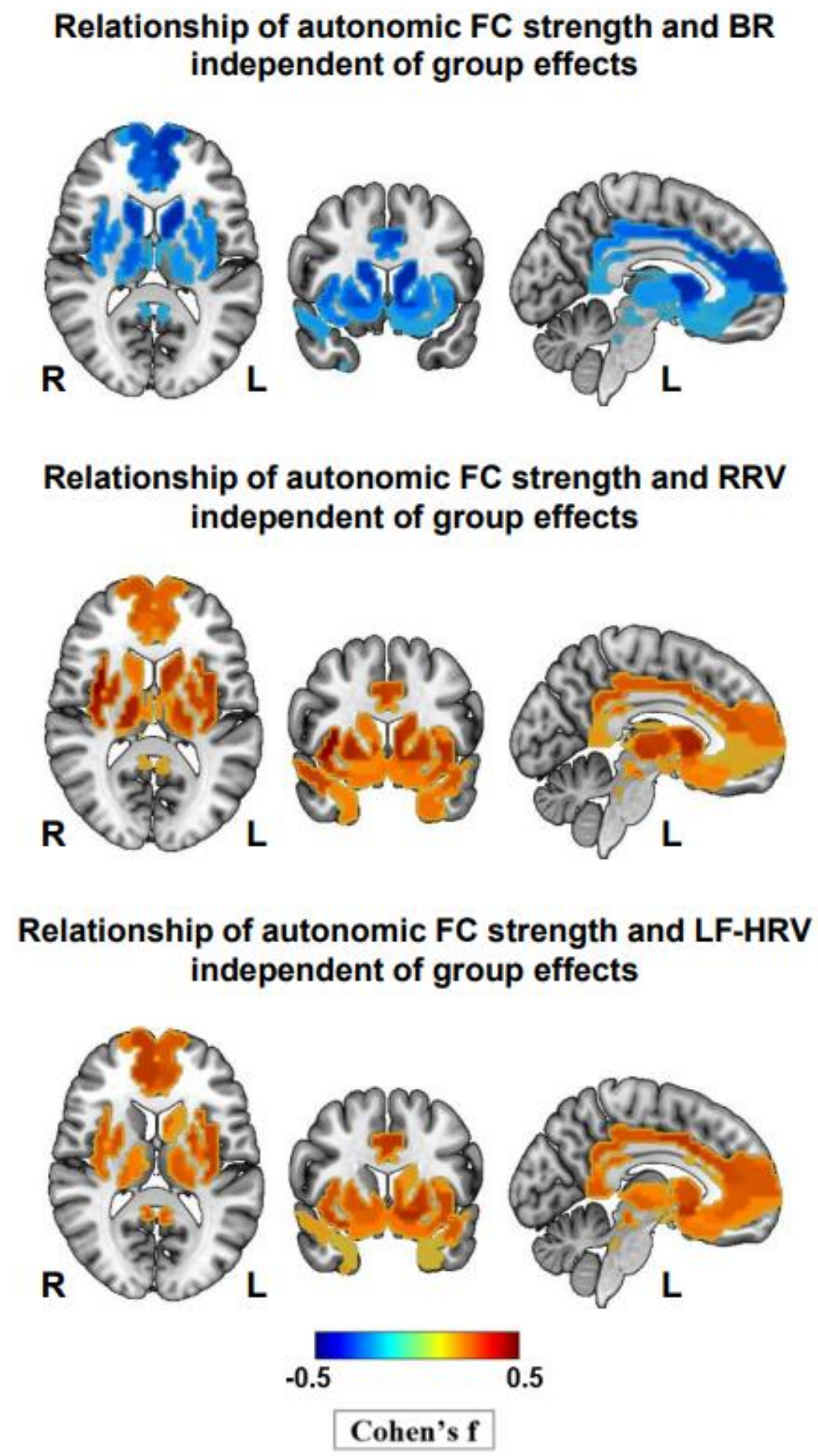

eFigure 3. Regional associations between the functional connectivity (FC) node strength of the central autonomic network with breathing rate (BR), respiratory rate variability (RRV), and low-frequency heart rate variability (LF-HRV), independent of group effects (healthy control or temporal lobe epilepsy) ($p < 0.05$, false discovery rate [FDR]-corrected across regions). General linear models (GLMs) were used to test for a significant moderation effect of group on the relationship between FC and cardiorespiratory measures. The effect size (Cohen's *f*) was derived for the FC term in the GLMs and weighted by the sign of beta coefficient to indicate directionality. The FC and cardiorespiratory estimates were adjusted for age and sex.

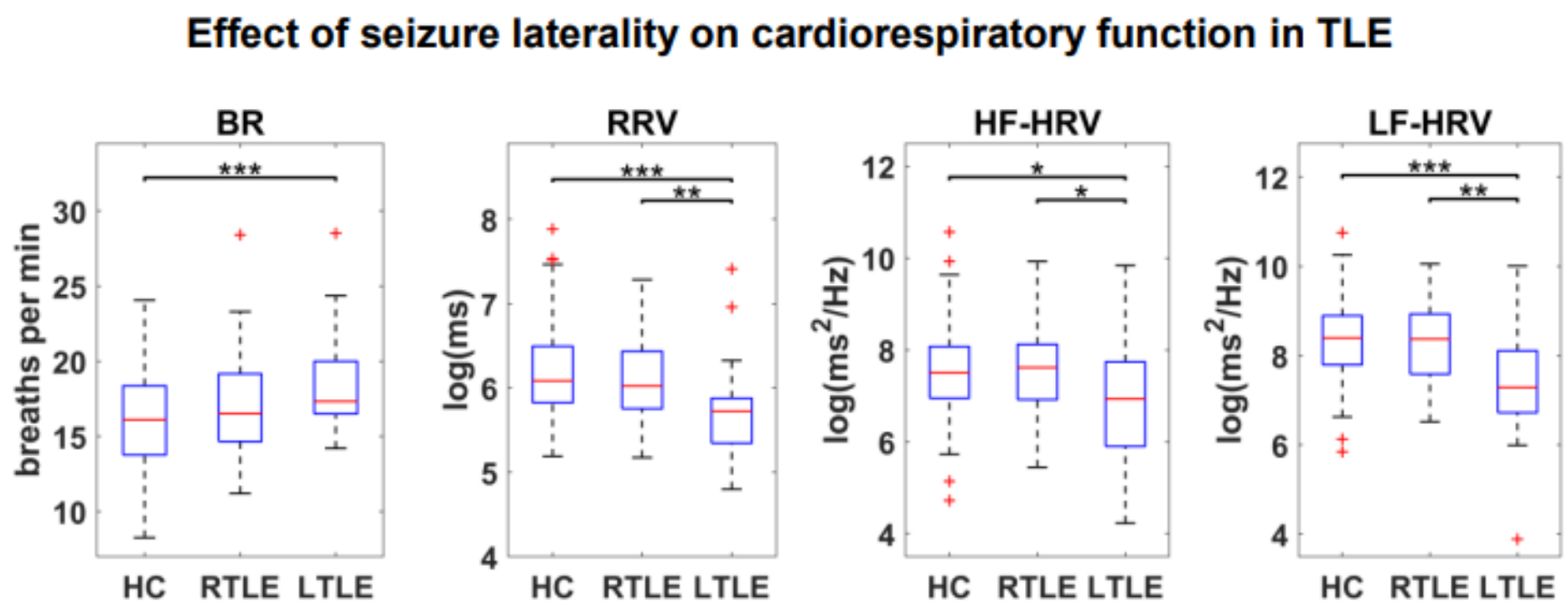

eFigure 4. Pairwise comparisons of the breathing rate (BR), respiratory rate variability (RRV), and high- and low-frequency heart rate variability (HF/LF-HRV) between healthy controls (HC), right temporal lobe epilepsy (RTLE), and left temporal lobe epilepsy (LTLE). Two-samples *t*-tests (two-tailed) were used for statistical comparisons. *$p < 0.05$, **$p < 0.01$, ***$p < 0.001$, false discovery rate (FDR)-corrected across four cardiorespiratory measures and three pairwise comparisons. The cardiorespiratory estimates were adjusted for age and sex.

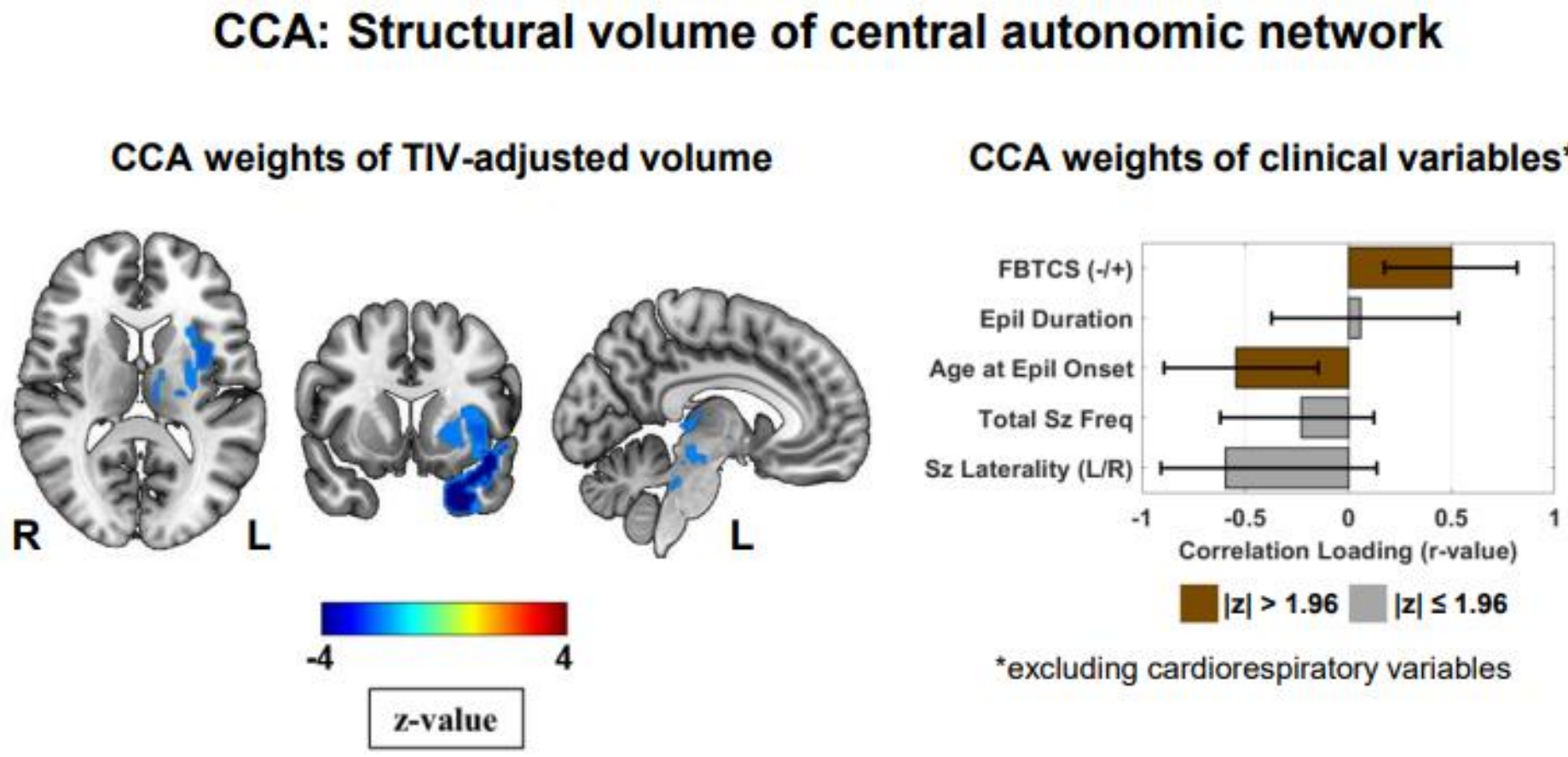

eFigure 5. Loading weights of the CCA mode that maximized the correlation between the structural volume of the central autonomic network with the clinical parameters in temporal lobe epilepsy, while adjusting the brain volume estimates for total intracranial volume (TIV) ($r = 0.66$; $p = 0.019$, family-wise error rate [FWER]-corrected across five modes; permutation test). Contribution of the volume of the central autonomic regions to the CCA mode ($|z| > 1.96$; bootstrap z-scores of the correlation loadings; *left brain plot*). Contribution of the clinical parameters to the CCA mode (correlation loadings; *right bar plot*). Error bars correspond to 95% bootstrap confidence intervals. The brain volume estimates were also adjusted for age and sex.

eTable 3. Central autonomic regions with reduced FC node strength in TLE relative to controls, and the relationship between FC node strength and measures of cardiorespiratory function in TLE.

Brain Area	FC of HC < TLE (*t*-value, mean)[a]	FC vs BR in TLE (*rho*, mean)[b]	FC vs RRV in TLE (*rho*, mean)	FC vs LF-HRV in TLE (*rho*, mean)
**Left Hipp**	-3.46	N.S.	0.36	0.43
Right Hipp	-3.33	N.S.	N.S.	N.S.
Right Amyg	-2.23	N.S.	N.S.	N.S.
**Left Parahipp**	-3.02	N.S.	0.34	0.33
**Right Parahipp**	-3.06	N.S.	0.30	N.S.
**Left Fusiform**	-2.89	-0.32	0.29	0.33
**Right Fusiform**	-3.03	-0.33	0.33	0.29
**Left Temp Pole**	-3.65	N.S.	0.27	0.31
Right Temp Pole	-3.52	N.S.	N.S.	N.S.
**Left Insula**	-3.04	-0.32	0.34	0.35
**Right Insula**	-2.75	-0.32	0.35	0.33
**Left Cingulate**	-3.60	-0.39	0.32	0.31
**Right Cingulate**	-3.70	-0.36	0.34	0.32
**Left OFC**	-2.71	N.S.	N.S.	0.35
**Right OFC**	-2.52	N.S.	0.32	0.35
**Left Frontal Pole**	-3.37	-0.45	0.31	N.S.
**Right Frontal Pole**	-2.65	-0.37	0.27	0.27
**Left Basal Ganglia**	-3.68	-0.39	0.38	0.39
**Right Basal Ganglia**	-3.50	-0.40	0.36	0.34
**Left Thalamus**	-3.54	N.S.	N.S.	0.30
**Right Thalamus**	-2.93	N.S.	0.35	0.35
**mRt**	-2.25	N.S.	N.S.	0.32
**PBC**	-2.37	N.S.	0.33	0.34
PnO	-2.32	N.S.	N.S.	N.S.
PTg	-2.64	N.S.	N.S.	N.S.
VTA	-2.73	N.S.	N.S.	N.S.
**Left NBM**	-3.78	N.S.	0.33	N.S.
Right NBM	-2.74	N.S.	N.S.	N.S.
**Left BNST**	-2.91	N.S.	N.S.	0.32
Right BNST	-2.12	N.S.	N.S.	N.S.
Left HYP	-3.08	N.S.	N.S.	N.S.

FC = functional connectivity; TLE = temporal lobe epilepsy; HC = healthy control; BR = breathing rate; RRV = respiratory rate variability; LF-HRV = low-frequency heart rate variability; N.S. = not significant; Hipp = hippocampus; Amyg = amygdala; Parahipp = parahippocampal gyrus; Temp Pole = temporal pole; OFC = orbitofrontal cortex; mRt = mesencephalic reticular formation; PBC = parabrachial nuclear complex; PnO = pontine oralis; PTg = pedunculotegmental nucleus; VTA = ventral tegmental area; NBM = nucleus basalis of Meynert; BNST = bed nucleus of the stria terminalis; HYP = hypothalamus.

Regions of interest (ROIs) are grouped by brain area and are defined from the Brainnetome atlas (100 cortical and subcortical autonomic ROIs),[14] Harvard Ascending Arousal Network atlas (10 brainstem ROIs),[15] Jubrain Anatomy Toolbox (four basal forebrain ROIs),[16] and Neudorfer-Germann atlas (two hypothalamus and two bed nucleus of the stria terminalis [BNST] ROIs).[17]

Bold entries denote brain areas with significant associations between the FC node strength and at least one of the cardiorespiratory measures in TLE ($p < 0.05$, false discovery rate [FDR]-corrected across all autonomic ROIs).

a) The mean is computed across the ROIs in each brain area with significant differences in FC strength between TLE and controls ($p < 0.05$, FDR-corrected across all autonomic ROIs).

b) The mean is computed across the ROIs in each brain area with (i) significant differences in FC strength between TLE and controls and (ii) significant associations between FC strength and the indicated cardiorespiratory measure ($p < 0.05$, FDR-corrected across all autonomic ROIs).

eTable 4. Central autonomic regions in which structural volume was reduced in TLE relative to controls, with and without adjustment for total intracranial volume.

Brain Area	Volume of HC < TLE, without TIV adjustment (*t*-value, mean)[a]	Volume of HC < TLE, with TIV adjustment (*t*-value, mean)
Left Hipp	-4.26	-5.20
Right Hipp	-4.49	-5.09
Left Parahipp	-2.58	N.S.
Right Parahipp	-2.56	N.S.
Left Fusiform	-2.61	N.S.
Right Fusiform	-2.65	N.S.
Left Temp Pole	-2.56	N.S.
Right Temp Pole	-2.31	N.S.
Left Insula	-2.43	N.S.
Right Insula	-2.50	N.S.
Left Cingulate	-2.99	N.S.
Right Cingulate	-3.31	-3.03
Left OFC	-2.71	N.S.
Right OFC	-2.56	N.S.
Left Frontal Pole	-2.90	N.S.
Right Frontal Pole	-2.77	N.S.
Left Basal Ganglia	-4.14	-3.48
Right Basal Ganglia	-4.28	-4.09
Left Thalamus	-4.67	-4.20
Right Thalamus	-4.52	-4.02
mRt	-3.89	-3.25
PBC	-2.53	N.S.
PnO	-3.23	N.S.
VTA	-3.50	-2.65
Left NBM	-4.16	-3.45
Right NBM	-3.73	-2.77
Left HYP	-2.99	N.S.
Right HYP	-3.06	N.S.

TIV = total intracranial volume; TLE = temporal lobe epilepsy; N.S. = not significant; Hipp = hippocampus; Parahipp = parahippocampal gyrus; Temp Pole = temporal pole; OFC = orbitofrontal cortex; mRt = mesencephalic reticular formation; PBC = parabrachial nuclear complex; PnO = pontine oralis; VTA = ventral tegmental area; NBM = nucleus basalis of Meynert; HYP = hypothalamus.

Regions of interest (ROIs) are grouped by brain area and are defined from the Brainnetome atlas (100 cortical and subcortical autonomic ROIs),[14] Harvard Ascending Arousal Network atlas (10 brainstem ROIs),[15] Jubrain Anatomy Toolbox (four basal forebrain ROIs),[16] and Neudorfer-Germann atlas (two hypothalamus and two bed nucleus of the stria terminalis [BNST] ROIs).[17]

a) The mean is computed across the ROIs in each brain area with significant differences in structural volume between TLE and controls ($p < 0.05$, FDR-corrected across all ROIs).

eTable 5. Central autonomic regions in which FC node strength mediated differences in cardiorespiratory function between the two groups (TLE and controls).

Brain Area	FC mediation of group differences in BR (z-value, mean)[a]	FC mediation of group differences in RRV (z-value, mean)	FC mediation of group differences in LF-HRV (z-value, mean)
Left Hipp	3.09	-2.92	-3.53
Right Hipp	N.S.	-2.68	-2.59
Right Amyg	N.S.	N.S.	-2.15
Left Parahipp	2.40	-2.88	-2.87
Right Parahipp	3.10	-3.01	-2.98
Left Fusiform	3.05	-3.04	-3.09
Right Fusiform	3.12	-3.25	-3.47
Left Temp Pole	2.42	-3.45	-2.67
Right Temp Pole	2.92	-3.23	-2.61
Left Insula	3.12	-2.91	-2.89
Right Insula	2.94	-2.67	-2.66
Left Cingulate	3.40	-3.22	-3.22
Right Cingulate	3.35	-3.14	-3.25
Left OFC	2.64	-2.52	-2.63
Right OFC	2.58	-2.40	-2.41
Left Frontal Pole	3.48	-2.97	-3.29
Right Frontal Pole	2.79	-2.50	-2.48
Left Basal Ganglia	3.77	-3.37	-3.39
Right Basal Ganglia	3.53	-3.13	-3.36
Left Thalamus	2.90	-3.13	-3.12
Right Thalamus	2.93	-3.02	-2.98
mRt	2.37	N.S.	-2.14
PBC	2.62	-2.45	-2.36
PnO	2.70	-2.19	N.S.
PTg	2.35	-2.60	-2.45
VTA	2.86	-2.68	-2.35
Left NBM	4.02	-2.92	-2.95
Left BNST	2.88	-2.62	-2.59
Right BNST	2.32	N.S.	N.S.
Left HYP	2.71	-2.77	-2.34

FC = functional connectivity; TLE = temporal lobe epilepsy; BR = breathing rate; RRV = respiratory rate variability; LF-HRV = low-frequency heart rate variability; N.S. = not significant; Hipp = hippocampus; Amyg = amygdala; Parahipp = parahippocampal gyrus; Temp Pole = temporal pole; OFC = orbitofrontal cortex; mRt = mesencephalic reticular formation; PBC = parabrachial nuclear complex; PnO = pontine oralis; PTg = pedunculotegmental nucleus; VTA = ventral tegmental area; NBM = nucleus basalis of Meynert; BNST = bed nucleus of the stria terminalis; HYP = hypothalamus.

Regions of interest (ROIs) are grouped by brain area and are defined from the Brainnetome atlas (100 cortical and subcortical autonomic ROIs),[14] Harvard Ascending Arousal Network atlas (10 brainstem ROIs),[15] Jubrain Anatomy Toolbox (four basal forebrain ROIs),[16] and Neudorfer-Germann atlas (two hypothalamus and two bed nucleus of the stria terminalis [BNST] ROIs).[17]

a) For each brain area, the mean is computed across the ROIs in which FC node strength significantly mediated group differences in the indicated cardiorespiratory measure ($p < 0.05$, false discovery rate [FDR]-corrected across all autonomic ROIs).

eTable 6. Associations between cardiorespiratory measures and clinical parameters after matching patients with left- and right-lateralized TLE based on propensity scores (*n* = 31).

Multivariate models (multiple linear regression)[a]								
	**BR**		**RRV**		**HF-HRV**		**LF-HRV**	
	**Cohen's *f***	P_{FDR}	**Cohen's *f***	P_{FDR}	**Cohen's *f***	P_{FDR}	**Cohen's *f***	P_{FDR}
FBTCS occurrence (yes, ref: no)	-	-	-	-	-	-	-	-
Epilepsy duration	0.18	0.17	-	-	-	-	-	-
Age at epilepsy onset	-	-	-	-	-	-	0.23	0.12
Total seizure frequency	0.19	0.17	-	-	-	-	-	-
Seizure laterality (left, ref: right)	0.29	0.058	**-0.45**	**0.006**	**-0.31**	**0.044**	**-0.40**	**0.011**

TLE = temporal lobe epilepsy; BR = breathing rate; RRV = respiratory rate variability; HF-HRV = high-frequency heart rate variability; LF-HRV = low-frequency heart rate variability; FBTCS = focal-to-bilateral tonic-clonic seizures.

Bold entries denote statistically significant associations at $p < 0.05$, false discovery rate (FDR)-corrected across cardiorespiratory measures and clinical parameters.

a) A rank-based inverse normal transformation was applied to ensure normality of the residuals, and LASSO regression with leave-one-out cross-validation was used for variable selection. The effect size (Cohen's f) is weighted by the sign of the beta coefficient to indicate directionality.

eTable 7. Correlation loading weights of central autonomic regions that reliably contributed to the significant CCA modes derived from FC node strength, non-TIV-adjusted volume, and TIV-adjusted volume in TLE.

**Brain Area**	**CCA loadings of FC node strength (*r*-value, mean)[a]**	**CCA loadings of non-TIV-adjusted volume (*r*-value, mean)**	**CCA loadings of TIV-adjusted volume (*r*-value, mean)**
Left Hipp	-0.49	**-0.70**	**-0.53**
Left Amyg	-0.57	**-0.79**	**-0.61**
Right Amyg	-0.56	-0.40	N.S.
Left Parahipp	**-0.55**	**-0.67**	**-0.57**
Right Parahipp	-0.53	-0.46	N.S.
Left Fusiform	-0.65	**-0.64**	**-0.73**
Right Fusiform	-0.68	-0.48	N.S.
Left Temp Pole	**-0.58**	**-0.80**	**-0.74**
Right Temp Pole	**-0.70**	-0.50	N.S.
Left Insula	**-0.66**	**-0.54**	-0.45
Right Insula	**-0.71**	N.S.	N.S.
Left Cingulate	-0.67	-0.40	N.S.
Right Cingulate	**-0.69**	-0.39	N.S.
Left OFC	**-0.62**	-0.53	-0.41
Right OFC	**-0.59**	N.S.	N.S.
Left Frontal Pole	-0.54	N.S.	N.S.
Right Frontal Pole	**-0.68**	N.S.	N.S.
Left Basal Ganglia	**-0.70**	-0.51	-0.41
Right Basal Ganglia	**-0.72**	N.S.	N.S.
Left Thalamus	**-0.75**	**-0.54**	-0.49
Right Thalamus	**-0.81**	N.S.	N.S.
DR	N.S.	-0.46	N.S.
LC	-0.48	**-0.60**	-0.42
LDTg	N.S.	-0.41	N.S.
mRt	**-0.73**	**-0.60**	-0.43
MnR	**-0.67**	-0.43	N.S.
PAG	-0.54	N.S.	N.S.
PBC	**-0.80**	**-0.62**	-0.51
PnO	**-0.72**	**-0.57**	-0.45
PTg	-0.49	**-0.58**	-0.45
VTA	-0.49	**-0.61**	-0.45
Left MS/DBB	-0.42	-0.40	N.S.
Left NBM	N.S.	**-0.56**	N.S.
Left BNST	-0.54	N.S.	N.S.
Right BNST	-0.45	N.S.	N.S.
Right HYP	-0.41	N.S.	N.S.

CCA = canonical correlation analysis; FC = functional connectivity; TIV = total intracranial volume; TLE = temporal lobe epilepsy; N.S. = not significant; Hipp = hippocampus; Amyg = amygdala; Parahipp = parahippocampal gyrus; Temp Pole = temporal pole; OFC = orbitofrontal cortex; DR = dorsal raphe; LC = locus coeruleus; LDTg = laterodorsal tegmental nucleus; mRt = mesencephalic reticular formation; MnR = median raphe; PAG = periaqueductal gray; PBC = parabrachial nuclear complex; PnO = pontine oralis; PTg = pedunculotegmental nucleus; VTA = ventral tegmental area; MS/DBB = medial septum/diagonal band of Broca; NBM = nucleus basalis of Meynert; BNST = bed nucleus of the stria terminalis; HYP = hypothalamus.

Regions of interest (ROIs) are grouped by brain area and are defined from the Brainnetome atlas (100 cortical and subcortical autonomic ROIs),[14] Harvard Ascending Arousal Network atlas (10 brainstem ROIs),[15] Jubrain Anatomy Toolbox (four basal forebrain ROIs),[16] and Neudorfer-Germann atlas (two hypothalamus and two bed nucleus of the stria terminalis [BNST] ROIs).[17]

For each CCA mode, bold entries denote brain areas that include ROIs with the most reliable loadings ($z < -3$).

a) For each brain area, the mean is computed across ROIs with reliable loadings in the CCA modes at bootstrap z-score < -1.96.

eTable 8. Comparison of cardiorespiratory measures between patients with and without postsurgical MRI.

	***r*-value**	***P* uncorr.**	P_{FDR}
BR	0.09	0.46	0.61
RRV	0.04	0.71	0.71
HF-HRV	0.12	0.31	0.61
LF-HRV	0.09	0.44	0.61

BR = breathing rate; RRV = respiratory rate variability; HF-HRV = high-frequency heart rate variability; LF-HRV = low-frequency heart rate variability.

Wilcoxon rank-sum tests were used to compare each cardiorespiratory measure between patients with and without postsurgical MRI. False discovery rate (FDR) correction was performed across four cardiorespiratory measures.

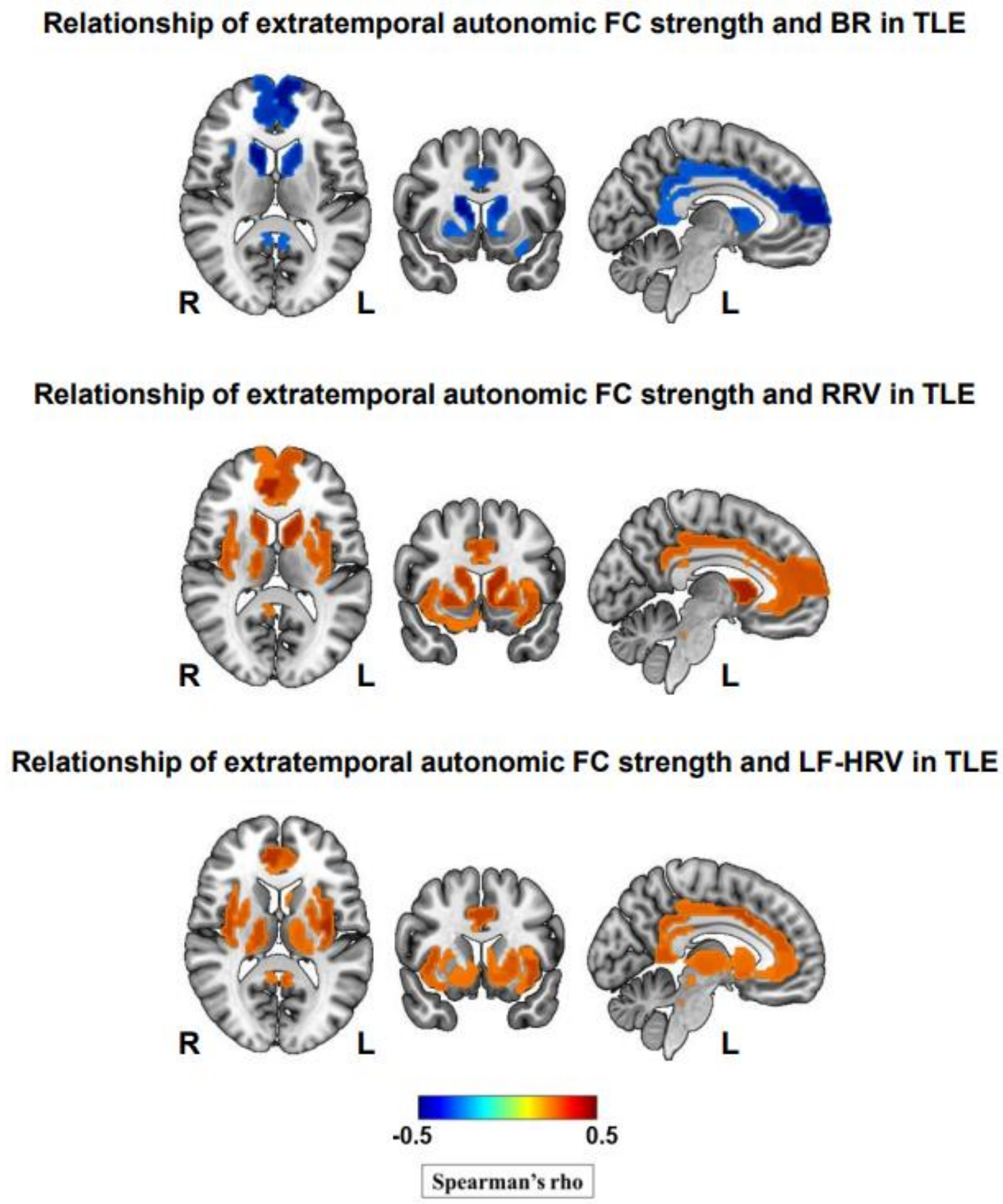

eFigure 6. Regional associations between the functional connectivity (FC) node strength of the extratemporal central autonomic network with breathing rate (BR), respiratory rate variability (RRV), and low-frequency heart rate variability (LF-HRV) in temporal lobe epilepsy (TLE) ($p < 0.05$, false discovery rate [FDR]-corrected across regions; Spearman correlation tests, two-tailed). The FC node strength was computed while excluding brain regions in the right and left temporal lobes. The FC and cardiorespiratory estimates were adjusted for age and sex.

eTable 9. Relationship between pre-to-postsurgical differences in each mean FC value and corresponding cardiorespiratory measure, while controlling for surgery type and time between surgery and postsurgical scan.

Partial Spearman correlation models						
	**BR**		**RRV**		**LF-HRV**	
	***rho* (95% CI)**	***P* uncorr.**	***rho* (95% CI)**	***P* uncorr.**	***rho* (95% CI)**	***P* uncorr.**
Mean FC	-0.15 (-0.52 to 0.23)	0.40[a]	**0.41 (-0.01 to 0.69)**	**0.019[b]**	**0.52 (0.18 to 0.74)**	**0.0025[c]**
Surgery type (ATL, ref: SAH/SLAH)	-0.18 (-0.50 to 0.19)	0.32	0.16 (-0.20 to 0.44)	0.39	-0.27 (-0.56 to 0.15)	0.13
Time after surgery	-0.01 (-0.39 to 0.33)	0.94	-0.14 (-0.47 to 0.31)	0.43	0.28 (-0.11 to 0.61)	0.12

BR = breathing rate; RRV = respiratory rate variability; LF-HRV = low-frequency heart rate variability; FC = functional connectivity; ATL = anterior temporal lobectomy; SAH = selective amygdalohippocampectomy; SLAH = selective laser amygdalohippocampectomy.

Bold entries denote statistically significant associations at $p < 0.05$, uncorrected. Bootstrapping ($n = 1 \times 10^5$) was used to derive 95% confidence intervals for Spearman's *rho* values.

a) $p = 0.40$, false discovery rate (FDR)-corrected across three cardiorespiratory measures.

b) $p = 0.029$, false discovery rate (FDR)-corrected across three cardiorespiratory measures.

c) $p = 0.0076$, false discovery rate (FDR)-corrected across three cardiorespiratory measures.

eTable 10. Relationship between pre-to-postsurgical differences in each mean FC value and corresponding cardiorespiratory measure in patients who underwent selective amygdalohippocampectomy.

Partial Spearman correlation models						
	**BR**		**RRV**		**LF-HRV**	
	***rho* (95% CI)**	***P* uncorr.**	***rho* (95% CI)**	***P* uncorr.**	***rho* (95% CI)**	***P* uncorr.**
Mean FC	-0.20 (-0.61 to 0.28)	0.35[a]	0.35 (-0.16 to 0.68)	0.096[b]	**0.46 (0.00 to 0.75)**	**0.025[c]**
Time after surgery	0.06 (-0.39 to 0.44)	0.78	-0.19 (-0.55 to 0.30)	0.38	0.19 (-0.29 to 0.61)	0.38

BR = breathing rate; RRV = respiratory rate variability; LF-HRV = low-frequency heart rate variability; FC = functional connectivity.

Bold entries denote statistically significant associations at $p < 0.05$, uncorrected. Bootstrapping ($n = 1 \times 10^5$) was used to derive 95% confidence intervals for Spearman's *rho* values.

a) $p = 0.35$, false discovery rate (FDR)-corrected across three cardiorespiratory measures.

b) $p = 0.14$, false discovery rate (FDR)-corrected across three cardiorespiratory measures.

c) $p = 0.076$, false discovery rate (FDR)-corrected across three cardiorespiratory measures.

eTable 11. Effect of clinical parameters on pre-to-postsurgical differences in each cardiorespiratory and mean FC measure.

Effect on cardiorespiratory measures (partial Spearman correlation models)						
	**BR**		**RRV**		**LF-HRV**	
	rho **(95% CI)**	***P*** **uncorr.**	***rho*** **(95% CI)**	***P*** **uncorr.**	***rho*** **(95% CI)**	***P*** **uncorr.**
Engel seizure outcome	0.17 (-0.19 to 0.48)	0.34	-0.26 (-0.58 to 0.15)	0.14	**-0.38 (-0.67 to 0.01)**	**0.033**
Surgery type (ATL, ref: SAH/SLAH)	-0.20 (-0.51 to 0.14)	0.26	0.20 (-0.15 to 0.51)	0.28	-0.18 (-0.51 to 0.14)	0.31
Time after surgery	-0.05 (-0.42 to 0.30)	0.78	-0.03 (-0.40 to 0.35)	0.88	**0.40 (0.04 to 0.68)**	**0.022**
**Effect on mean FC measures (partial Spearman correlation models)**						
	**Mean FC [BR]**		**Mean FC [RRV]**		**Mean FC [LF-HRV]**	
	rho **(95% CI)**	***P*** **uncorr.**	***rho*** **(95% CI)**	***P*** **uncorr.**	***rho*** **(95% CI)**	***P*** **uncorr.**
Engel seizure outcome	-0.12 (-0.46 to 0.22)	0.50	-0.08 (-0.41 to 0.25)	0.67	-0.13 (-0.45 to 0.20)	0.49
Surgery type (ATL, ref: SAH/SLAH)	-0.02 (-0.32 to 0.30)	0.90	0.02 (-0.32 to 0.34)	0.93	0.00 (-0.33 to 0.32)	1.00
Time after surgery	0.06 (-0.30 to 0.44)	0.73	0.15 (-0.22 to 0.52)	0.42	0.19 (-0.17 to 0.54)	0.29

BR = breathing rate; RRV = respiratory rate variability; LF-HRV = low-frequency heart rate variability; ATL = anterior temporal lobectomy; SAH = selective amygdalohippocampectomy; SLAH = selective laser amygdalohippocampectomy; FC = functional connectivity.

Bold entries denote statistically significant associations at $p < 0.05$, uncorrected. Bootstrapping ($n = 1 \times 10^5$) was used to derive 95% confidence intervals for Spearman's *rho* values.

eTable 12. Effect of current number of AEDs and MRI evidence of MTS on cardiorespiratory measures in TLE.

Pairwise analyses						
	**Number of AEDs[a]**			**MRI evidence of MTS[b]**		
	rho	***P* uncorr.**	**P_{FDR}**	***r*-value**	***P* uncorr.**	**P_{FDR}**
BR	0.00	1.00	1.00	0.00	0.99	0.99
RRV	-0.05	0.65	0.86	-0.01	0.95	0.99
HF-HRV	-0.23	0.051	0.10	-0.16	0.18	0.54
LF-HRV	**-0.28**	**0.017**	**0.066**	-0.13	0.27	0.54

TLE = temporal lobe epilepsy; BR = breathing rate; RRV = respiratory rate variability; HF-HRV = high-frequency heart rate variability; LF-HRV = low-frequency heart rate variability; AED = antiepileptic drug; MTS = mesial temporal sclerosis.

False discovery rate (FDR) correction was performed across four cardiorespiratory measures. Bold entries denote statistically significant associations at $p < 0.05$, uncorrected.

a) Spearman's correlation tests between number of AEDs per patient and each cardiorespiratory measure.

b) Wilcoxon rank-sum tests comparing each cardiorespiratory measure between patients with and without MRI evidence of MTS.

eTable 13. Reliability of cardiorespiratory measures across the two fMRI scans per participant.

	**All participants**		**Patients with TLE**		**Healthy controls**	
	**ICC (95% CI)**	**Cohen's *d***	**ICC (95% CI)**	**Cohen's *d***	**ICC (95% CI)**	**Cohen's *d***
BR	0.87 (0.78 to 0.92)	0.07	0.88 (0.75 to 0.94)	-0.03	0.86 (0.72 to 0.92)	0.14
RRV	0.82 (0.75 to 0.87)	0.04	0.79 (0.66 to 0.87)	0.14	0.82 (0.73 to 0.88)	-0.02
HF-HRV	0.92 (0.89 to 0.94)	0.34	0.93 (0.89 to 0.96)	0.43	0.91 (0.86 to 0.93)	0.28
LF-HRV	0.87 (0.83 to 0.91)	0.41	0.90 (0.85 to 0.95)	0.37	0.83 (0.77 to 0.88)	0.43

TLE = temporal lobe epilepsy; ICC = intra-class correlation coefficient; BR = breathing rate; RRV = respiratory rate variability; HF-HRV = high-frequency heart rate variability; LF-HRV = low-frequency heart rate variability.

Bootstrapping ($n = 1 \times 10^5$) was used to derive 95% confidence intervals for ICC values.

eReferences

1. Gonzalez HFJ, Narasimhan S, Johnson GW, et al. Role of the Nucleus Basalis as a Key Network Node in Temporal Lobe Epilepsy. Neurology 2021;96:e1334-e1346.
2. Gonzalez HFJ, Narasimhan S, Goodale SE, et al. Arousal and salience network connectivity alterations in surgical temporal lobe epilepsy. J Neurosurg 2023;138:810-820.
3. Glover GH, Li TQ, Ress D. Image-based method for retrospective correction of physiological motion effects in fMRI: RETROICOR. Magn Reson Med 2000;44:162-167.
4. Pourmotabbed H, Martin CG, Goodale SE, et al. Multimodal state-dependent connectivity analysis of arousal and autonomic centers in the brainstem and basal forebrain. Imaging Neurosci (Camb) 2025;3.
5. Beissner F. Functional MRI of the Brainstem: Common Problems and their Solutions. Clin Neuroradiol 2015;25 Suppl 2:251-257.
6. Chang C, Metzger CD, Glover GH, Duyn JH, Heinze HJ, Walter M. Association between heart rate variability and fluctuations in resting-state functional connectivity. Neuroimage 2013;68:93-104.
7. Quigley KS, Gianaros PJ, Norman GJ, Jennings JR, Berntson GG, de Geus EJC. Publication guidelines for human heart rate and heart rate variability studies in psychophysiology-Part 1: Physiological underpinnings and foundations of measurement. Psychophysiology 2024;61:e14604.
8. Makowski D, Pham T, Lau ZJ, et al. NeuroKit2: A Python toolbox for neurophysiological signal processing. Behav Res Methods 2021;53:1689-1696.
9. Magana-Tellez O, Maganti R, Hupp NJ, et al. Sleep EEG and respiratory biomarkers of sudden unexpected death in epilepsy (SUDEP): a case-control study. Lancet Neurol 2025;24:840-849.
10. Stuart EA. Matching methods for causal inference: A review and a look forward. Stat Sci 2010;25:1-21.
11. Pourmotabbed H, de Jongh Curry AL, Clarke DF, Tyler-Kabara EC, Babajani-Feremi A. Reproducibility of graph measures derived from resting-state MEG functional connectivity metrics in sensor and source spaces. Hum Brain Mapp 2022;43:1342-1357.
12. Lu K, Sjors Dahlman A, Karlsson J, Candefjord S. Detecting driver fatigue using heart rate variability: A systematic review. Accid Anal Prev 2022;178:106830.
13. Boudreau P, Yeh WH, Dumont GA, Boivin DB. Circadian variation of heart rate variability across sleep stages. Sleep 2013;36:1919-1928.

14. Fan L, Li H, Zhuo J, et al. The Human Brainnetome Atlas: A New Brain Atlas Based on Connectional Architecture. Cereb Cortex 2016;26:3508-3526.

15. Edlow BL, Olchanyi M, Freeman HJ, et al. Multimodal MRI reveals brainstem connections that sustain wakefulness in human consciousness. Sci Transl Med 2024;16:eadj4303.

16. Zaborszky L, Hoemke L, Mohlberg H, Schleicher A, Amunts K, Zilles K. Stereotaxic probabilistic maps of the magnocellular cell groups in human basal forebrain. Neuroimage 2008;42:1127-1141.

17. Neudorfer C, Germann J, Elias GJB, Gramer R, Boutet A, Lozano AM. A high-resolution in vivo magnetic resonance imaging atlas of the human hypothalamic region. Sci Data 2020;7:305.
